# A multi-tissue atlas of regulatory variants in cattle

**DOI:** 10.1038/s41588-022-01153-5

**Published:** 2022-08-11

**Authors:** Shuli Liu, Yahui Gao, Oriol Canela-Xandri, Sheng Wang, Ying Yu, Wentao Cai, Bingjie Li, Ruidong Xiang, Amanda J. Chamberlain, Erola Pairo-Castineira, Kenton D’Mellow, Konrad Rawlik, Charley Xia, Yuelin Yao, Pau Navarro, Dominique Rocha, Xiujin Li, Ze Yan, Congjun Li, Benjamin D. Rosen, Curtis P. Van Tassell, Paul M. Vanraden, Shengli Zhang, Li Ma, John B. Cole, George E. Liu, Albert Tenesa, Lingzhao Fang

**Affiliations:** 1 Animal Genomics and Improvement Laboratory, Henry A. Wallace Beltsville Agricultural Research Center, Agricultural Research Service, USDA, Beltsville, Maryland 20705, USA; 2 The Roslin Institute, Royal (Dick) School of Veterinary Studies, The University of Edinburgh, Midlothian EH25 9RG, UK; 3 MRC Human Genetics Unit at the Institute of Genetics and Cancer, The University of Edinburgh, Edinburgh EH4 2XU, UK; 4 College of Animal Science and Technology, China Agricultural University, Beijing 100193, China; 5 Department of Animal and Avian Sciences, University of Maryland, College Park, Maryland 20742, USA; 6 State Key Laboratory of Genetic Resources and Evolution, Kunming Institute of Zoology, Chinese Academy of Sciences, Kunming, Yunnan 650223, China; 7 Institute of Animal Science, Chinese Academy of Agricultural Science, Beijing 100193, China; 8 Scotland’s Rural College (SRUC), Roslin Institute Building, Midlothian EH25 9RG, UK; 9 Faculty of Veterinary & Agricultural Science, The University of Melbourne, Parkville 3052, Victoria, Australia; 10 Agriculture Victoria, AgriBio, Centre for AgriBiosciences, Bundoora, Victoria 3083, Australia; 11 INRAE, AgroParisTech, GABI, Université Paris-Saclay, Jouy-en-Josas, F-78350, France; 12 Guangdong Provincial Key Laboratory of Waterfowl Healthy Breeding, College of Animal Science & Technology, Zhongkai University of Agriculture and Engineering, Guangzhou, Guangdong 510225, China; 13 School of Life Sciences, Westlake University, Hangzhou, Zhejiang 310024, China

**Keywords:** Cattle, expression QTLs, GWAS, RNA-Seq, TWAS

## Abstract

Characterization of genetic regulatory variants acting on the livestock gene expression is essential for interpreting the molecular mechanisms underlying traits of economic value and for increasing the rate of genetic gain through artificial selection. Here we build a Cattle Genotype-Tissue Expression atlas (CattleGTEx) as part of the pilot phase of Farm animal GTEx (FarmGTEx) project for the research community based on publicly available 7,180 RNA-Seq samples. We describe the transcriptomic landscape of over 100 tissues/cell types and report hundreds of thousands of genetic associations with gene expression and alternative splicing for 23 distinct tissues. We evaluate the tissue-sharing patterns of these genetic regulatory effects, and functionally annotate them using multi-omics data. Finally, we link gene expression in different tissues to 43 economically important traits using both transcriptome-wide association and colocalization analyses to decipher the molecular regulatory mechanisms underpinning such agronomic traits in cattle.

## Introduction

Genome-wide association studies (GWAS) have identified thousands of genetic variants associated with complex traits in human and livestock populations^1,2^. As the majority of these variants are non-coding, the characterization of molecular mechanisms by which such variants affect complex traits has been extremely challenging. Indeed, in human genetics, projects such as the Genotype-Tissue Expression (GTEx) project that have characterized genetic effects on the human transcriptome and paved the way to understanding the molecular mechanisms of human variation^3^.

However, livestock genomic resources lag behind human genomic resources, and to date, no study has systematically explored the regulatory variants of transcriptome across a wide range of tissues. GWAS signals of agronomic traits are significantly enriched in regulatory regions of genes expressed in trait-relevant tissues in cattle^4-6^, but studies of genetic variation in gene expression have generally been small, both in terms of the number of individuals and tissues. For instance, previous studies have explored the expression/splicing quantitative trait loci (e/sQTL) in blood^7^, milk cells^7^, muscle^8^ and mammary gland in cattle^9^.

There has been a recent exponential growth in the number of RNA-Seq samples made publicly available in cattle (Extended Data Figure 1a), but these data have never been uniformly processed and jointly analyzed before. Here, we present a pipeline to uniformly integrate 7,180 public RNA-Seq samples, representing over 100 different tissues and cell types, and identify eQTLs and sQTLs for 23 distinct cattle tissues with sufficient sample sizes (n > 40). The latter is facilitated by calling variants directly from the RNA-Seq reads and imputing to sequence level using a large multi-breed reference panel^10^, in a similar process to that used with human data^11^. Next, we conducted *in silico* analyses to annotate eQTLs and sQTLs with a variety of omics data in cattle, including DNA methylation, chromatin states, and chromatin conformation characteristics. Finally, we integrated gene expression with a large GWAS of 27,214 dairy bulls and 43 cattle traits *via* both transcriptome-wide association study (TWAS) and colocalization analyses to detect genes and variants associated with these economically important traits. We make the results freely and easily accessible to the research community through a web portal (http://cgtex.roslin.ed.ac.uk/). This Cattle Genotype-Tissue Expression (CattleGTEx) atlas as part of the Farm animal GTEx (FarmGTEx) project will serve as a primary reference for cattle genomics, breeding, adaptive evolution, veterinary medicine, and comparative genomics.

## Results

### Data summary

We analyzed 8,653 public RNA-Seq samples, yielding ~200 billion clean reads. The details of data summary are shown in Extended Data Figure 1b-i and Supplementary Table 1. We kept 7,180 samples with sufficient quality (see Methods) for subsequent analyses, representing 114 tissues from 46 breeds and breed combinations. Holstein was the most represented breed (35.5% of all samples), reflecting its global economic value. A total of 1,831 samples (21%) had no breed records, but that information could be inferred from the genotypes called from RNA-Seq data. We grouped the 114 tissues into 13 categories based on known biology and the 46 breeds into six ancestry groups, with *Bos taurus* representing 87% of all samples (Supplementary Table 1). To investigate the tissue-specificity of DNA methylation for functionally annotating QTLs, we also uniformly analyzed 144 whole-genome bisulfite sequence (WGBS) samples from 21 cattle tissues, producing ~73 billion clean reads with an average mapping rate of 71% (Supplementary Table 2).

### General characteristics of transcriptome across samples

As expected, the number of expressed genes (Transcripts per Million, TPM > 0.1) increased with the number of clean reads across samples. However, we observed a plateau at 50 million clean reads (Extended Data Figure 2a) where we only detected ~60% of 27,607 Ensembl annotated genes. Only 61 genes were not expressed in any of the samples, and 33 of them (54.10%) were located in unplaced scaffolds, with significantly shorter gene length, fewer exons, higher CG density, and lower sequence constraints than expressed genes (Extended Data Figure 2b-f). Similarly, we detected more alternative splicing events with increasing numbers of clean reads across samples (Extended Data Figure 2g). However, we did not detect splicing events for 874 genes in any sample, which also exhibited significantly shorter gene length, fewer exons, lower expression, and lower sequence constraints than spliced genes (Extended Data Figure 2h-k). Furthermore, 27% of them were snRNAs, snoRNAs and rRNAs that play important roles in RNA splicing^12^ (Extended Data Figure 2l). Genes without splicing events were significantly enriched in the integral component of membrane and G-protein coupled receptor signaling pathways (Extended Data Figure 2m). We found that ~25% of CpG sites in the entire genome were not covered at 5× in any of the WGBS samples, even if these had more than 300 million clean reads, partially due to bisulfite treatment and PCR amplification bias (Extended Data Figure 3a). These CpG sites were enriched in gene deserts (e.g., telomeres) with significantly higher CG density than the CpG sites captured by the WGBS (Extended Data Figure 3b-c).

We called a median of 21,623 SNPs from all RNA-Seq samples (Extended Data Figure 4a), and then imputed each sample up to 3,824,444 SNPs using a multi-breed reference population of 3,310 animals^10^. We validated the imputation accuracy by comparing SNPs derived from RNA-Seq with those called from whole-genome sequence (WGS) in the same individuals, including Holstein, Limousin and Angus breeds, and the concordance rates were over 99% (Extended Data Figure 4b, and Supplementary Table 3). We also compared the imputed genotypes from RNA-Seq data with those imputed using 50K SNP array genotypes in 109 Holstein animals. Although there was a depletion of high-quality (DR^2^ > 0.80) imputed intergenic variants amongst SNPs imputed from RNA-Seq only (Extended Data Figure 4c), the imputation accuracy of SNPs from RNA-Seq were similar to those from SNP-array along 1Mb up-/down- stream of gene body (Extended Data Figure 4d). In addition, the correlation of genotype counts between imputed SNPs from RNA-Seq data and those from SNP array was around 0.80 (Extended Data Figure 4e). For the subsequent *cis*-QTL mapping, we focused on 23 distinct tissues with greater than 40 individuals after removing duplicated samples within each tissue (Extended Data Figure 4f), and this encompassed 4,889 samples.

**Figure 1:**
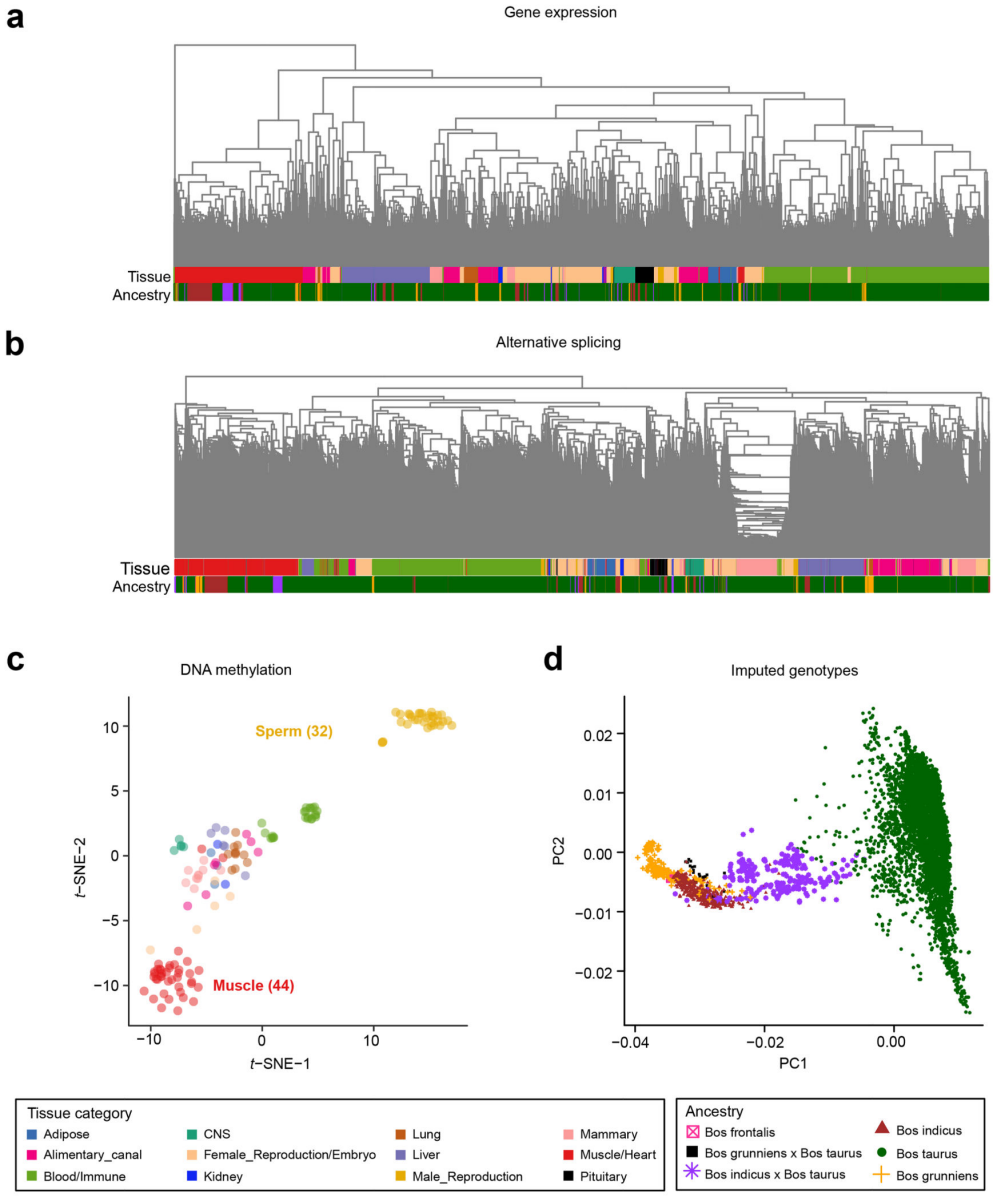
Hierarchical clustering and principal component analysis of samples. **(a)** Sample (n = 7,180) hierarchical clustering based on expression levels of all transcribed genes (Transcripts Per Million, TPM > 0.1). **(b)** Sample (n = 7,180) hierarchical clustering based on alternative splicing value (Percent Spliced-In, PSI) of spliced introns. **(c)** Sample (n = 144) clustering using *t*-distributed SNE coordinates based on DNA methylation levels of CpG sites (coverage ≥ 5×). **(d)** Principal component analysis of samples (n = 7,180) based on imputed genotypes.

We found that clusters of samples derived from both gene expression and alternative splicing could accurately recapitulated tissue types (Figure 1a, b), reinforcing the quality and therefore their utility for our follow-up analysis. For instance, all the muscle samples from over 40 projects clustered together. Similar to expression and splicing, DNA methylation profiles also recapitulated tissue types (Figure 1c). When clustering based on imputed genotypes, as expected, samples clustered by ancestry (Figure 1d).

### Tissue specificity of transcriptome and methylome

**Figure 2:**
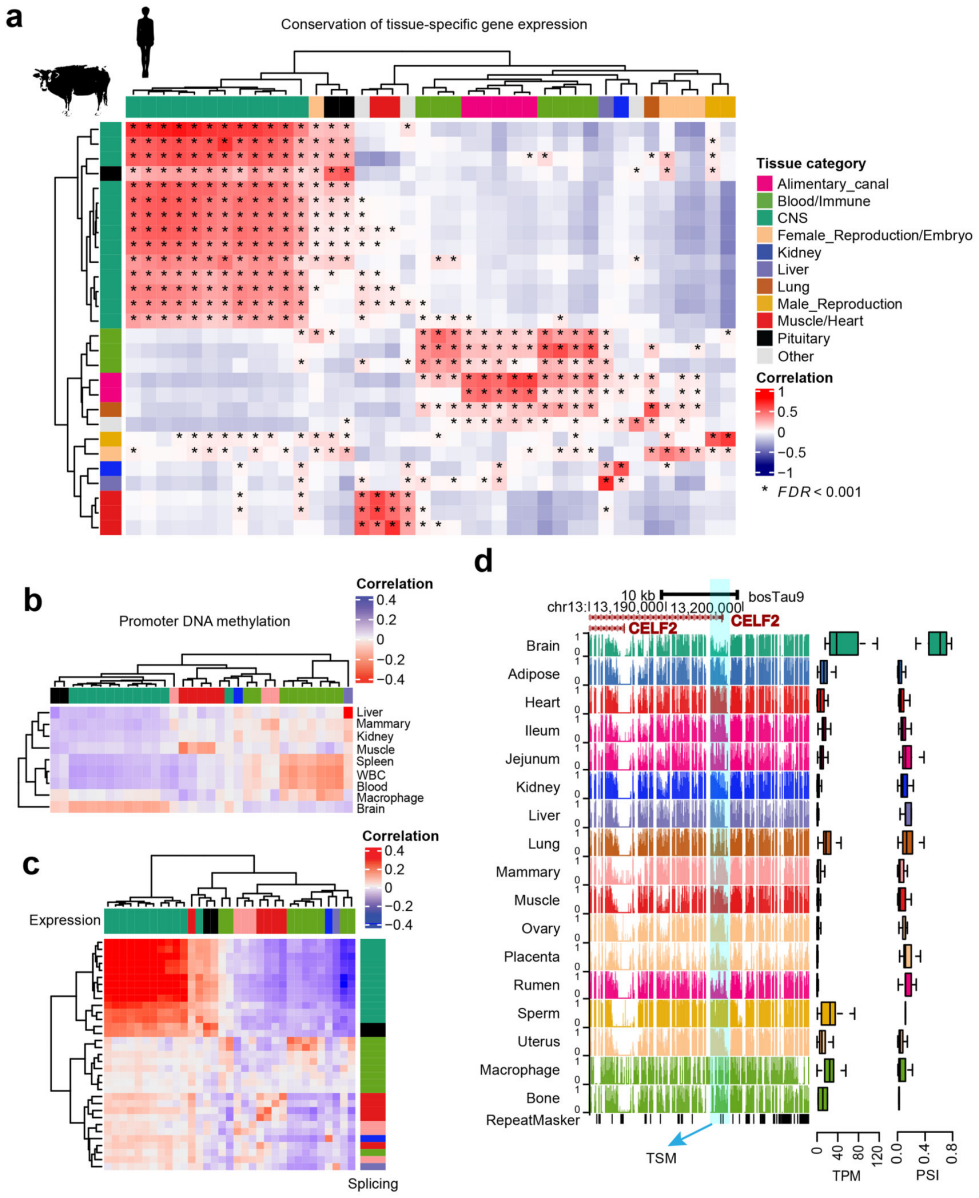
Tissue-specificity of gene expression, alternative splicing and DNA methylation. **(a)** Pearson correlation of tissue-specificity (measured as *t*-statistics) of 22,752 orthologous genes between cattle and human tissues (GTEx v8)^3^. The multiple testing is corrected for using Benjamini-Hochberg method (i.e., FDR). * denotes FDR < 0.001. **(b)** Pearson correlation of tissue-specificity between gene expression (*x*-axis) and promoter DNA methylation levels (*y*-axis). WBC is for white blood cells. The color code of tissues in *x*-axis is the same as that in (**a)**. **(c)** Pearson correlation of tissue-specificity between gene expression (Transcripts per Million, TPM, *x*-axis) and alternative splicing (Percent Spliced-In, PSI, *y*-axis). The color code of tissues is the same as that in (**a)**. **(d)** *CELF2* shows lower DNA methylation levels in splice sites (right), higher gene expression (middle), and higher PSI value of spliced introns (left) in brain tissue (n = 15) compared to the rest of tissues. TSM is for tissue-specific methylation.

Tissue-specificity of gene expression was significantly conserved between cattle and humans (Figure 2a), and the function of genes with tissue-specific expression accurately reflected the known biology of tissues. For instance, brain-specific genes were significantly enriched for synapse and neuron function, and testis-specific genes for spermatogenesis and reproduction (Extended Data Figure 5a). We also calculated tissue-specificity of promoter DNA methylation and gene alternative splicing. Similarly, the function of genes with tissue-specific promoter hypomethylation and splicing reflected the known biology of tissues (Extended Data Figure 5b-c). We found that, based on tissue-specificity, the gene expression level was significantly and negatively correlated with DNA methylation level in promoters (Figure 2b), and positively correlated with splicing ratios of introns (Figure 2c). For example, *CELF2*, a brain-related gene, had a significantly higher expression, lower promoter DNA methylation, and higher splicing ratio of first intron in brain than in other tissues considered (Figure 2d). Tissue-specific genes exhibited distinct patterns of sequence constraints (Extended Data Figure 5d), supporting the hypothesis of tissue-driven genome evolution^4^. We found that while brain-specific genes evolve slowly, blood or testis-specific ones evolve rapidly. This trend was also observed within tissue-specific hypomethylated regions (Extended Data Figure 5e-f).

### Discovery of expression and splicing QTLs

**Figure 3:**
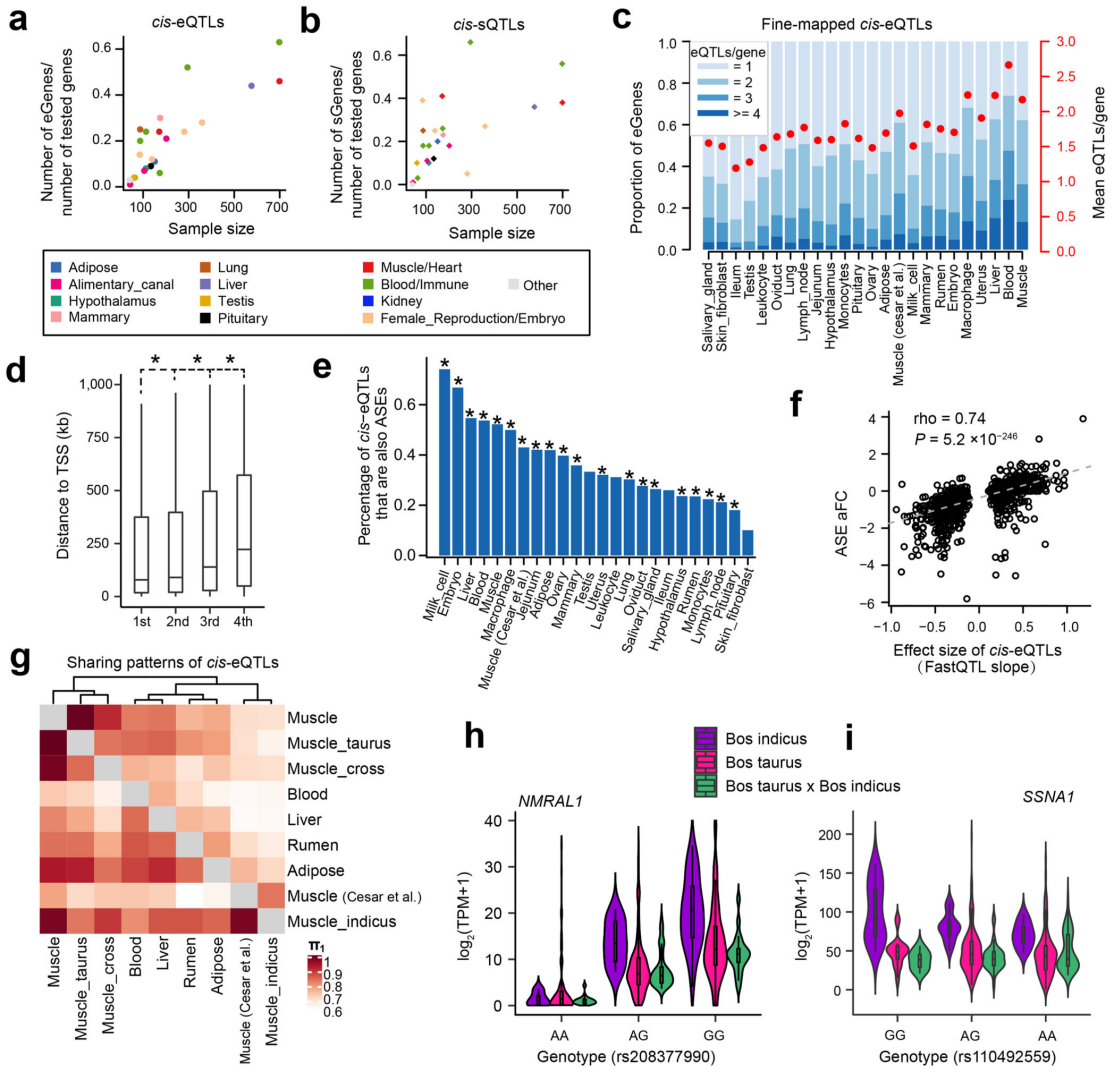
Discovery and characterization of *cis*-eQTLs and *cis*-sQTLs. **(a)** Relationship between the percentages of eGenes over all tested genes and sample size (Pearson *r* = 0.85; the two-sided Student’s *t*-test: *P* = 1.30×10^-7^) across 23 distinct tissues. **(b)** Relationship between the percentage of sGenes over all tested genes and sample size (Pearson *r* = 0.63; the two-sided Student’s *t*-test: *P* = 1.06×10^-3^) across 23 distinct tissues. Tissues are colored according to their tissue categories. **(c)** Distribution and average number of conditionally independent eQTLs per gene across tissues. Tissues are ordered by sample size. **(d)** The distance to Transcription Start Site (TSS) increases from the 1^st^ to 4^th^ independent eQTLs. Only 7276 gene-tissue pairs with at least 4 independent eQTLs were chosen. Significant differences (denoted as *) were observed between 1^st^ vs. 2^nd^ (*P* = 2.4×10^-3^), 2^nd^ vs. 3^rd^ (*P* = 3.0 ×10^-26^) and 3^rd^ vs. 4^th^ (*P* = 1.9×10^-27^) independent eQTLs based on the two-sided paired sample *t*-test. **(e)** *cis*-eQTLs are significantly (*P* < 1.0×10^-14^, denoted as *, Fisher Exact test) overrepresented in the loci with allelic specific expression (ASE). The *y*-axis indicates the percentage of *cis*-eQTLs that are also ASEs over all tested SNPs in the ASE analysis. **(f)** Correlation of effect sizes (FastQTL slope) of *cis*-eQTLs and allelic fold change (aFC) of ASEs (Spearman’s rho = 0.74, the two-sided Student’s *t*-test: *P* = 5.2×10^-246^) in liver. (**g**) Pairwise *cis*-eQTL sharing patterns (π1 value) of muscle tissue across three breeds (*Bos indicus*, *Bos taurus* and their crosses) and other tissues. Rows are discovery tissues, and columns are validation tissues. Muscle (Cesar et al.) is for 160 skeletal muscle samples of *Bos indicus* downloaded from Cesar et al. 2018^8^. **(h)** A *cis*-eQTL (rs208377990) of *NMRAL1* in muscle is shared across *Bos indicus* (n = 51), *Bos taurus* (n = 505) and their crosses (n = 108). **(i)** A *cis*-eQTL (rs110492559) of *SSNA1* in muscle is specific in *Bos indicus* (MAF = 0.25 and 0.37 in *Bos taurus* and *Bos indicus*, respectively), and has a significant (the two-sided *t*-test, *P* = 5.61×10^-3^) genotype × breed interaction. The samples are the same as in **(h)**.

We identified *cis*-e/sQTLs for 23 distinct tissues with 40 or more individuals, while accounting for relevant confounding factors and multiple testing (Extended Data Figure 6a-b). The number of eGenes (genes with significant *cis*-eQTLs) discovered ranged from 172 in ileum to 10,157 in blood, with 19,559 (83% of all 23,523 tested genes) classed as eGenes in at least one tissue (Supplementary Table 4). The number of sGenes (genes with significant *cis*-sQTLs) discovered ranged from four in the salivary gland to 7,913 in macrophages, with 15,376 (70.8%) classed as sGenes in at least one tissue. Genes with no *cis*-eQTLs or -sQTLs in any of the tissues were significantly enriched in hormone activity, regulation of receptor activity, neuropeptide signaling pathway, and reproduction (Supplementary Table 5-7). In general, the larger the number of samples for the tissue, the larger the number of *cis*-e/sGenes detected (Figure 3a-b). As expected, with a larger sample size, we had more power to detect *cis*-eQTLs with smaller effect sizes (Extended Data Figure 6c-d). Consistent with findings in humans^13^, significant variants (eVariants) centered around transcript start sites (TSS) of measured genes (Extended Data Figure 6e-f). Across 23 distinct tissues, an average of 46% (range 25.5 - 76.6%) of eVariants were found within 100 kb around TSS of target genes. In non-eGenes, there was also an enrichment of SNPs with the smallest *P*-values (but not statistically significant at FDR of 0.05) around TSS, suggesting a lack of power to detect such associations for those genes (Extended Data Figure 6e). Furthermore, we fine-mapped eGenes to assess whether the identified signals could be attributed to one or more causal SNPs. We found that an average of 46% (range 14.5 - 73.9%) of eGenes across 23 tissues had more than one independent *cis*-eQTLs (Figure 3c), indicating the complex genetic control of gene expression. SNPs with larger effects within a locus tended to be closer to the TSS (Figure 3d). To complement and validate the *cis*-eQTL analysis within individuals, we conducted an allele-specific expression (ASE) analysis, and found that *cis*-eQTLs were significantly overrepresented in loci with significant (FDR < 0.05) ASE (Figure 3e), and effect sizes of *cis*-eQTLs was significantly correlated with those of ASEs (Figure 3f, Extended Data Figure 6g).

To investigate whether *cis*-eQTLs are conserved among breeds, we conducted *cis*-eQTL mapping for muscle samples from *Bos indicus, Bos taurus*, and their hybrids separately, yielding 86, 2,766, and 800 eGenes, respectively. We observed that *cis*-eQTLs were more conserved across breeds than across tissues (Figure 3g). For example, the expression of *NMRAL1* in muscle was consistently and significantly regulated by a *cis*-eQTL (rs208377990) among *Bos indicus, Bos taurus*, and their hybrids (Figure 3h). Combining the summary statistics of each breed in a meta-analysis showed that eGene-eVariant associations identified in one breed are potentially transferable to other breeds, particularly for SNPs with larger effect size (Extended Data Figure 6h-i). Combining samples from different breeds will increase statistical power for detecting shared eQTLs, and enable more accurate mapping of the causal variants *via* reducing the linkage disequilibrium (LD) patterns. In total, 131 out of 437 eGene-eVariant pairs that were specifically discovered in *Bos indicus* showed significant (FDR < 0.05) genotype × breed interactions (Supplementary Table 8). For instance, the expression of an immune-related gene, *SSNA1*, was regulated by a *cis*-eQTL (rs110492559) in *Bos indicus* but not in *Bos taurus* or the hybrids, showing a significant genotype × breed interaction (Figure 3i). In addition, we found that breed-specific *cis*-eQTLs had lower minor allele frequency (MAF) than breed-common *cis*-eQTLs, consistent in both *Bos indicus* and *Bos taurus* (Extended Data Figure 7a-b). This may indicate that the difference in *cis*-eQTLs between breeds could be partially due to their difference in the frequency of segregating variants, provided that there are no epistatic/environmental/developmental effects.

**Figure 4:**
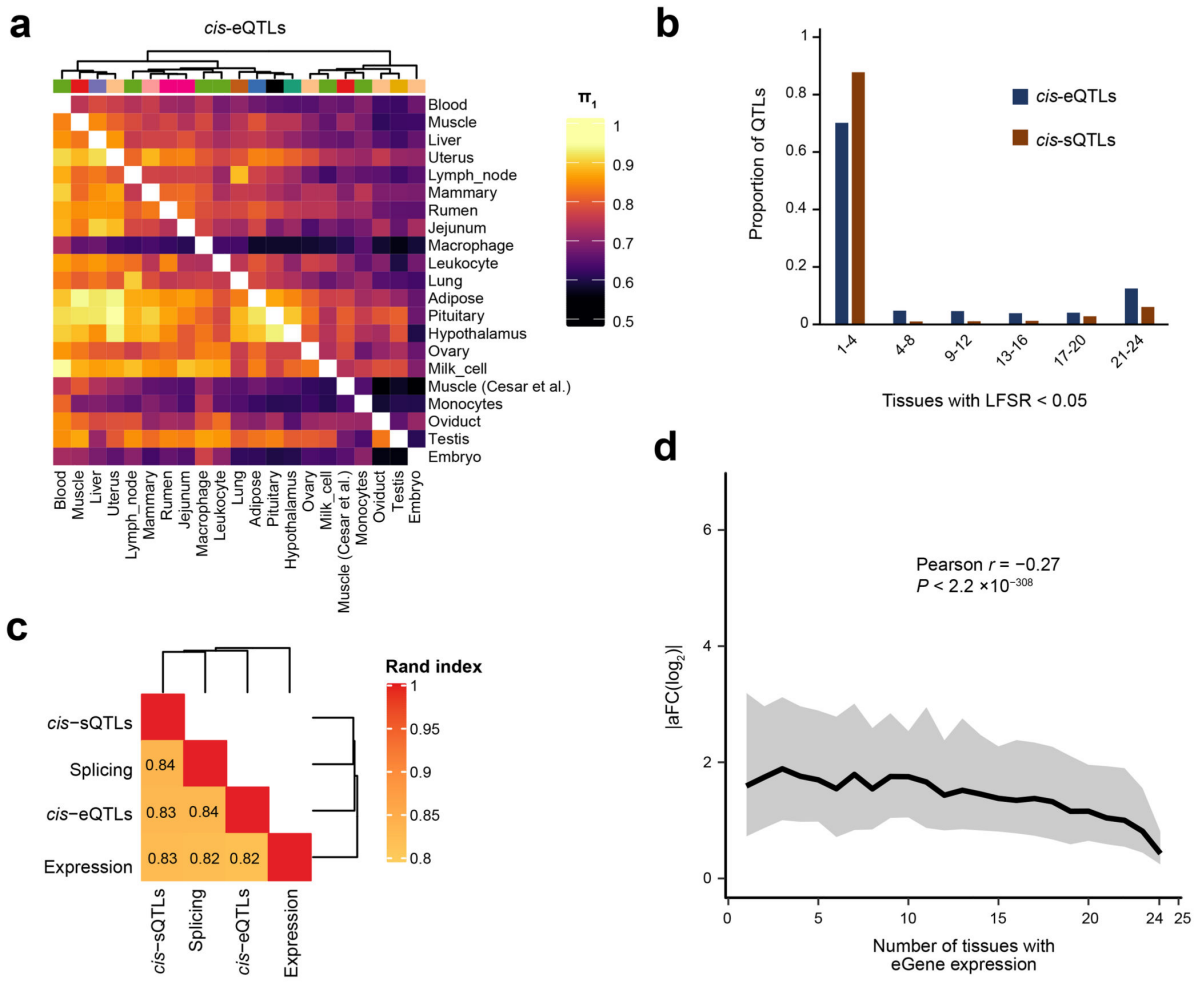
Tissue-sharing patterns of *cis*-QTLs. **(a)** Pairwise *cis*-eQTL sharing patterns (π1 value) across 23 distinct tissues. **(b)** Tissue activity of *cis*-eQTLs and *cis*-sQTLs, where a *cis*-QTL is considered active in a tissue if it has a *mashr* local false sign rate (LFSR, equivalent to FDR) of < 5%. **(c)** The similarity of tissue clustering across four data types (*cis*-eQTL, *cis*-sQTL, gene expression and splicing) based on the π1 values^3,13^. The k-means clustering is performed based on 2-22 clusters with 100,000 iterations. For each pairwise data types, we report the median Pairwise Rand index across all clusters. **(d)** Median (line) and 95% confidence interval (shading) of *cis*-eQTL effect size (*y*-axis, measured as the absolute log_2_ transformed allele Fold Change, |aFC(log2)|), as a function of the number of tissues in which the eGene is expressed (*x*-axis; TPM > 0.1). Pearson correlation between |aFC(log_2_)| and number of tissues with eGene expression is -0.27 (the two-sided Student’s *t*-test: df = 43,721; *P* < 2.2×10^-308^).

The tissue-sharing patterns of *cis*-QTLs could provide novel insights into molecular regulatory mechanisms underlying complex phenotypes^3^. We applied the π1 statistics to measure the sharing patterns of *cis*-e/sQTLs between tissues (Figure 4a and Extended Data Figure 7c). In general, we observed that both *cis*-eQTLs and *cis*-sQTLs tended to be tissue-specific or ubiquitous across tissues (Figure 4b). We also calculated the tissue-sharing patterns of gene expression and alternative splicing (Extended Data Figure 7d-e), and found that the tissue-sharing patterns of the four core data types (i.e., gene expression, alternative splicing and cis-e/sQTLs) were similar (Figure 4c and Extended Data Figure 7f). This result suggests that tissues with similar transcriptional profiles shared the genetic regulatory mechanisms of transcription. Further analysis on the expression of eGenes across tissues revealed that effect sizes of eVariants decreased with the increasing number of tissues where target eGenes were expressed, indicating that, on average, tissue-specific genes might be regulated by SNPs with larger genetic regulatory effects than widely-expressed genes (Figure 4d). Due to limitations and challenges of *trans-eQTLs* analysis in this study, which include: insufficient statistical power, the relatively lower imputation accuracy of distant intergenic SNPs, and complex inter-chromosomal LD in cattle (which could lead to increased type I error rates)^14^, we only conducted an exploratory *trans*-e/sQTL mapping for 15 tissues with over 100 individuals. We detected an average of 1,058 and 84 *trans-eGenes* and *trans*-sGenes (FDR < 0.05) across tissues, respectively (Supplementary Table 9). We summarized the details of *trans-eQTL* mapping, including LD patterns of *trans*-eQTLs and *cis*-eQTLs, tissue-sharing patterns of *trans*-eQTLs and their validations, in Extended Data Figure 8.

### Functional annotation of QTLs

**Figure 5:**
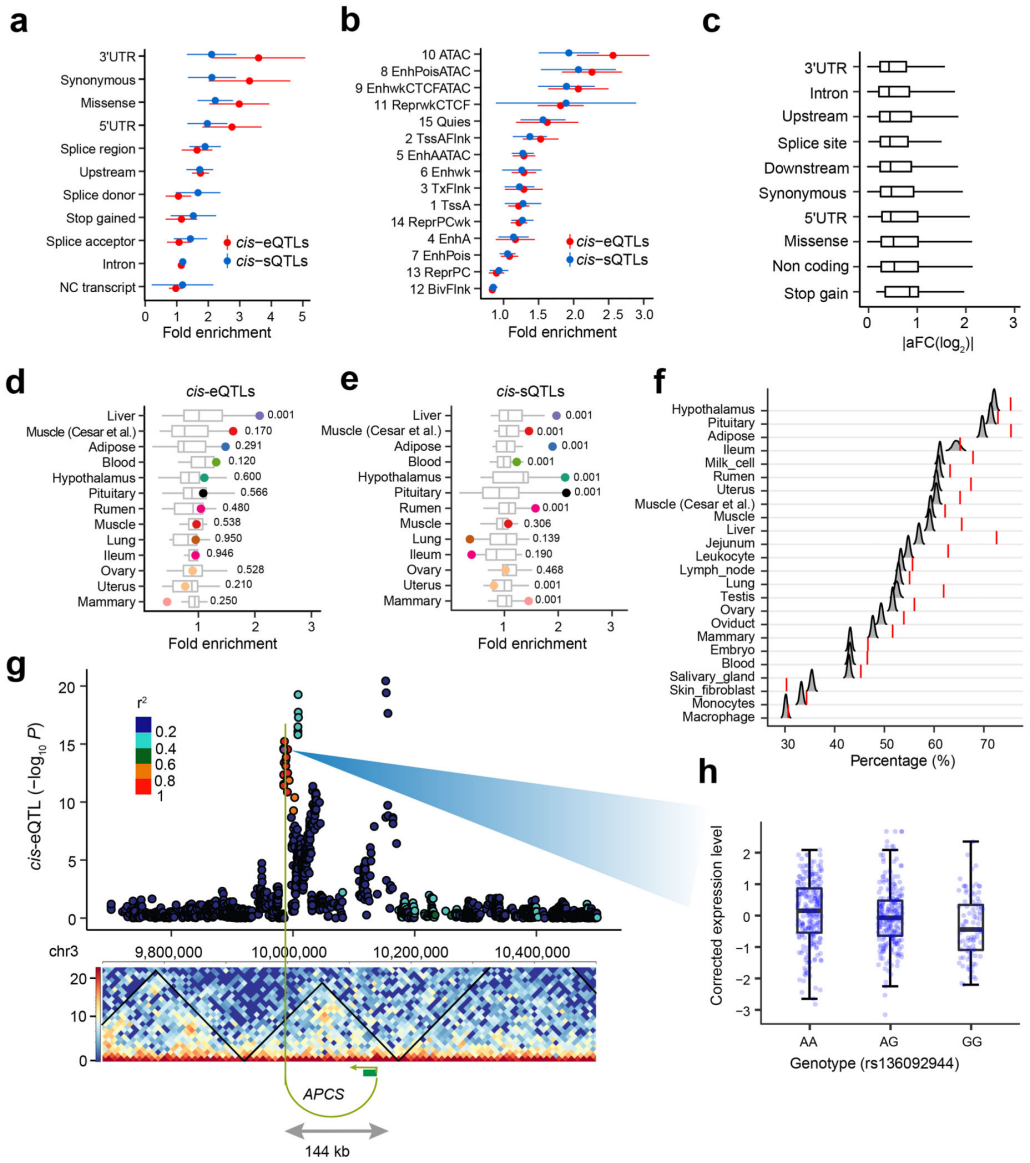
Functional annotation of *cis*-QTLs. **(a)** Enrichment (fold change, the two-sided permutation test with 1,000 times) of *cis*-eQTLs and *cis*-sQTLs of 23 distinct tissues in sequence ontology. The data are presented as Mean ± SD. **(b)** Enrichment (fold change, the two-sided permutation test with 1,000 times) of *cis*-eQTLs and *cis*-sQTLs of 23 distinct tissues in 15 chromatin states predicted from cattle rumen epithelial primary cells in Holstein animals^15^. The data are presented as Mean ± SD. **(c)** Effect sizes (measured as |aFC(log2|) of *cis*-eQTLs of 23 distinct tissues across sequence ontology. **(d)** and **(e)** Enrichment of *cis*-eQTLs and *cis*-sQTLs of 13 tissues in tissue-specific hypomethylated regions, respectively. These 13 tissues have both DNA methylation and *cis*-QTL data. The numbers are *P*-values for enrichments of matched tissues (highlighted dots) based on the permutation test (the two sided, 1,000 times). **(f)** Percentages of eGene-eVariant pairs that are located within topologically associating domains (TADs) are significantly (FDR < 0.01, one-sided) higher than those of random eGene-SNP pairs with matched distances, except for ileum, macrophage and skin fibroblast. The null distributions of percentages of eGene-SNP pairs within TADs are obtained by doing 5,000 bootstraps. The TADs are obtained from the lung Hi-C data. **(g)** An eGene (*APCS*) and its eVariant (rs136092944) are located within a TAD, and linked by a significant Hi-C contact (10kb bins, position 9985,000 is linked to 10,135,000 in chr3 with Benjamini-Hochberg corrected *P* = 1.4×10^-6^. The *P*-value is obtained based on the binominal distribution model. The Manhattan plot shows the *P*-values of all tested SNPs in the *cis*-eQTL mapping analysis of *APCS*. The linkage disequilibrium (LD, *r*^2^) values between eVariant (rs136092944) and surrounding SNPs are shown in colors. (**h**) The boxplot shows the PEER-corrected expression levels of *APCS* across the three genotypes of eVariant (rs136092944), i.e., AA (n = 237), AG (n = 245), and GG (n = 94), respectively.

We employed multiple layers of biological data to better define the molecular mechanisms of genetic regulatory effects. As expected, *cis*-e/sQTLs were significantly (*P* < 0.05, the 1,000 times permutation test) enriched in functional elements, such as 3’UTR and open chromatin regions by ATAC-Seq data in cattle rumen epithelial primary cells^15^ (Figure 5a-b). The *cis*-sQTLs had a higher enrichment in splice donors/acceptors than *cis*-eQTLs. The *cis*-eQTLs associated with stop gains had larger effect sizes than other *cis*-eQTLs (Figure 5c). The *cis*-e/sQTLs were enriched in hypomethylated regions of the matching tissues across 13 tissues (Figure 5d-e). For instance, the liver exhibited the highest enrichment of *cis*-e/sQTL in liver-specific hypomethylated regions. Consistent with the brain having distinct abundance of alternative splicing, related to the development of the nervous system^13^, *cis*-sQTLs in the hypothalamus and pituitary had the highest enrichments in their specific hypomethylated regions (Figure 5e).

Topologically associated domains (TADs) enable chromatin interactions between distant regulatory regions and target promoters^16^. By examining Hi-C data of lung tissue in cattle^17^, we obtained TADs and significant Hi-C contacts, which were likely to be conserved across tissues^16^. By comparing with random eGene-SNP pairs with matched distances, we observed significantly (FDR < 0.01, 5,000 bootstrapping test) higher percentages of eGene-eVariant pairs within TADs across the majority of tissues, except for ileum and skin fibroblast (Figure 5f). For instance, *APCS* and its *cis*-eQTL peak (144kb upstream of its TSS) were encompassed by a TAD and linked by a significant Hi-C contact, which allowed the regulation of its expression by a distant eVariant (rs136092944) (Figure 5g-h).

### *cis*-QTLs and complex trait associations

**Figure 6:**
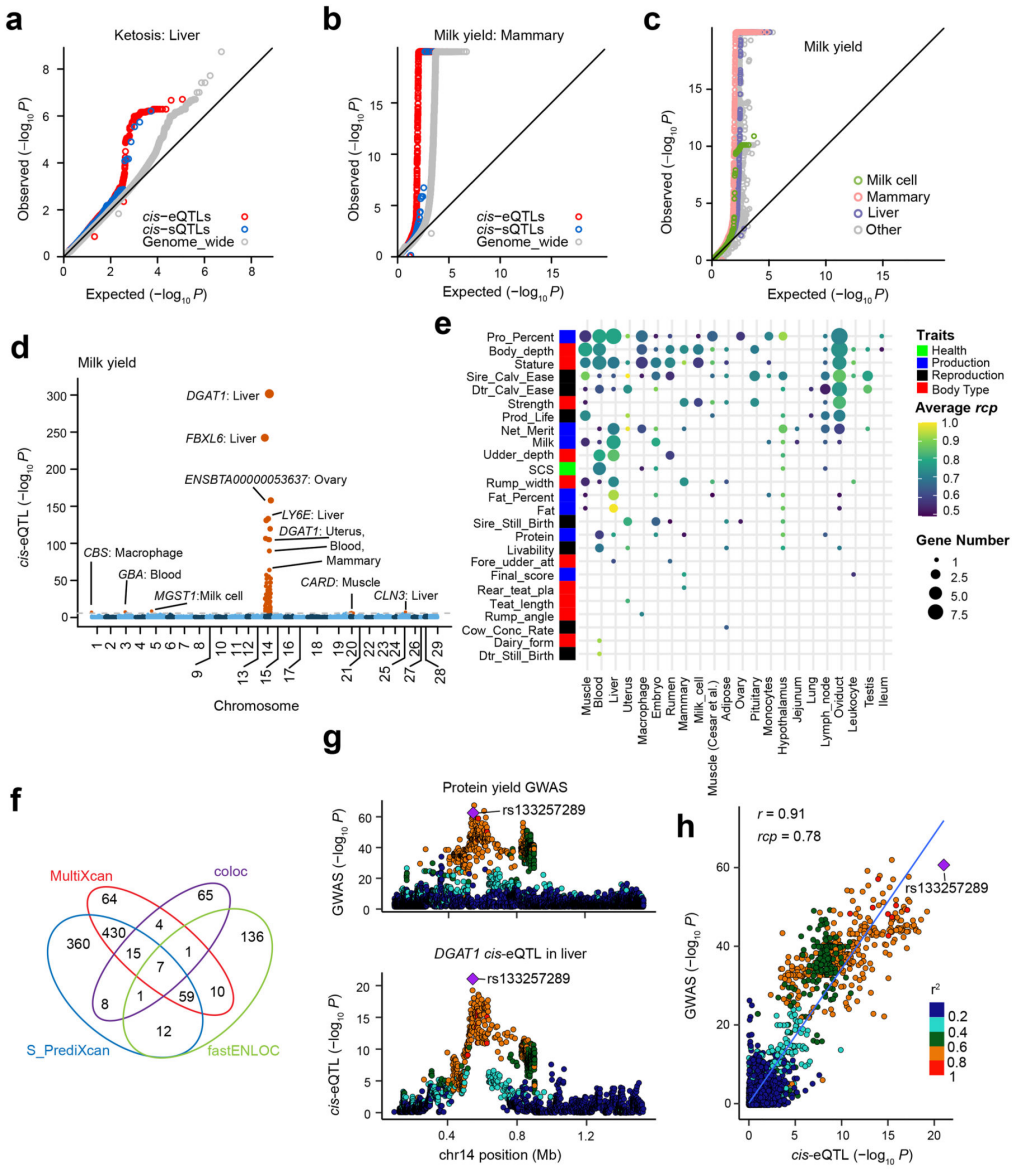
Relationship between complex traits and *cis*-QTLs. **(a)** *cis*-eQTLs (*P* = 0.001) and *cis*-sQTLs (*P* = 0.02) in liver show significantly higher enrichments for top SNPs associated with ketosis compared to genome-wide SNPs (shown in grey). **(b)** *cis*-eQTLs (*P* = 0.001) and *cis*-sQTLs (*P* = 0.03) in mammary gland show higher enrichments for top SNPs associated with milk yield compared to genome-wide SNPs (shown in grey). All the *P* values above are obtained by the two-sided permutation test with 1,000 times. **(c)** Enrichment of *cis*-eQTLs for genetic associations with milk yield is tissue-dependent. The *cis*-eQTLs in mammary gland, milk cells and liver exhibit higher enrichments for genetic associations with milk yield compared to those in other tissues. **(d)** Manhattan plots of transcriptome-wide association study (TWAS) for milk yield across all 23 distinct tissues. **(e)** The number of genes that were colocalized (regional colocalization probability, *rcp* > 0.5 in fastENLOC) between GWAS significant loci of complex traits and *cis*-eQTLs across tissues. The size of point indicates the number of genes, while the color of point indicates the average *rcp* of each trait-tissue pair. The abbreviations of GWAS traits are explained in Supplementary Table 10. **(f)** The overlaps of significant gene-trait pairs from TWAS with S-PrediXcan (Bonferroni corrected *P* < 0.05) and S-MultiXcan (Bonferroni corrected *P* < 0.05) and colocalization with fastENLOC (*rcp* > 0.5) and Coloc (posterior probability of the shared single causal variant hypothesis H4 (PP.H4) > 0.8). **(g)** An example of a colocalization (*rcp* = 0.78) of *cis*-eQTLs of *DGAT1* gene in liver and GWAS loci of protein yield in cattle on chromosome 14. The top colocalized SNP (rs133257289) is the top *cis*-eQTL of *DGAT1* and the second top GWAS signal of protein yield. **(h)** A high Pearson correlation (*r* = 0.91, the two-sided Student’s *t*-test: df = 2,933; *P* < 2.2×10^-308^) between *P*-values from *cis*-eQTLs of *DGAT1* in liver and GWAS of protein yield.

The primary goal of this study is to provide a resource for elucidating the genetic and biological mechanisms involved in cattle complex traits. We thus evaluated *cis*-e/sQTLs detected in each tissue for associations with four distinct agronomic traits as examples, i.e., ketosis, milk yield, age at first calving (AFC) and somatic cell score (SCS). The top SNPs associated with ketosis from GWAS were significantly (*P* < 0.05, the 1,000 times permutation test) enriched for liver *cis*-e/sQTLs (Figure 6a). Similarly, milk yield associated SNPs were significantly overrepresented in *cis*-e/sQTLs from mammary gland (Figure 6b). Compared to other tissues, mammary gland, milk cells and liver were the tissues with highest enrichment of milk yield associated SNPs amongst *cis*-eQTLs (Figure 6c). Additionally, SNPs associated with AFC were significantly enriched for monocytes *cis*-eQTLs, and SCS for mammary gland (Extended Data Figure 9a). We observed that a larger sample size of a *cis*-eQTL tissue resulted in a higher enrichment of GWAS loci and *cis*-eQTLs, potentially explaining the associations of complex traits with non-matching tissues (Extended Data Figure 9b).

We detected 854 significant gene-trait pairs for 43 agronomic traits (Supplementary Table 10) in cattle *via* single-tissue TWAS (S-PrediXcan), representing 337 unique genes (Supplementary Table 11). Out of 319 fine-mapped genes^18,19^, we validated 54, including linking expression of *DGAT1* in liver and mammary gland, and expression of *MGST1* in milk cells, as well as expression of *CLN3* in liver to milk yield (Figure 6d). The expression of *ZNF613* in hypothalamus was the most significant association for many reproduction and body conformation traits, including daughter-still-birth and stature (Supplementary Table 11), supporting our previous finding that *ZNF613* is significantly associated with gestation length possibly through its influence on embryonic development^20^. Furthermore, we conducted a colocalization analysis of *cis*-eQTLs and GWAS loci, and detected 115 unique eGenes that were colocalized (regional colocalization probability, *rcp* > 0.5) with 260 GWAS loci associated to 25 out of the 43 complex traits analyzed. These represented 235 significant gene-trait pairs (Figure 6e; Supplementary Table 12). For instance, *TIGAR*, a muscle *cis*-eGene, playing roles in the phosphatase activity, energy storage and consuming, was colocalized (*rcp* = 0.529) with one of independent GWAS signals of strength on chromosome 5 (Extended Data Figure 9c). GWAS loci of milk yield were colocalized with *ARHGAP39* in hypothalamus, *TEF* in embryo, *SYT11* in blood, *CCDC166* in oviduct and *ASPHD1* in jejunum (Supplementary Table 12). We also took sire calving ease, which GWAS loci were colocalized with 21 eGenes in at least one tissue, as an example in Extended Data Figure 9d. In addition, we further employed Coloc and S-MultiXcan to conduct the colocalization and multi-tissue TWAS analysis, and detected 110 and 590 significant gene-trait pairs, respectively (Supplementary Table 13-14). By comparing results from TWAS and colocalization, we found an overlap of seven gene-trait pairs (Figure 6f, Extended Data Figure 10). For instance, we found that *cis*-eQTLs of *DGAT1* in liver were colocalized (*rcp* = 0.78) with GWAS signals of protein yield, and the *p*-values from GWAS were highly (*r* = 0.91) correlated with those from *cis*-eQTL (Figure 6g-h).

## Discussion

The CattleGTEx atlas represents one of the most comprehensive reference resources of the cattle transcriptome to date. It provides a detailed characterization of genetic control of gene expression and splicing across 23 distinct tissues in cattle. This study demonstrates that it is possible to discover gene expression regulatory variants by deriving and imputing genetic variants from livestock RNA-Seq data alone. We established a *in silico* protocol to generate a livestock GTEx atlas in a timely manner and show the value of reanalyzing published data to find novel biology, avoiding the significant costs of data generation. Although we have provided a comprehensive view of the genetic regulatory variants in cattle, we are also mindful that this resource can be further improved with the inclusion of more individuals/breeds and further data types. The imputation accuracy for breeds that are very under-represented in the reference panel might be relatively low. Additionally, generating SNP genotypes or WGS for individuals with RNA-Seq data can provide additional information for distal intergenic variants as compared to RNA-Seq data only. The FarmGTEx consortium is currently extending the bioinformatics pipeline developed here to other livestock species (e.g., pig, sheep, goat and chicken).

The CattleGTEx also provides a resource to explore tissue-sharing patterns of the transcriptome and its genetic regulation in cattle. In contrast to the human GTEx^3^, where RNA-Seq samples across tissues were collected from the same individuals, the CattleGTEx used public data, where individuals or even breeds were different from tissue to tissue. This might explain why there is a lower proportion of *cis*-e/sQTLs shared across tissues compared to the human GTEx. In addition, the difference in the cell-type composition of tissues can also affect the tissue-sharing patterns of *cis*-QTLs^3^. When single-cell RNA-Seq data is available for multiple tissues in the near future^21^, it will be of interest to computationally estimate the cell-type proportions in the bulk-tissue samples to uncover the cellular specificity of genetic regulatory effects^22^.

This CattleGTEx atlas provides an important tool for studying the mechanisms underlying complex traits through systematically linking SNPs, genes, tissues, and complex traits. The e/sQTLs detected here provide a rich set of functional variants for agronomic traits in cattle, as we found that top GWAS associations of traits were significantly enriched for regulatory QTLs in their relevant tissues. Our TWAS and colocalization analyses further provide a list of promising candidate genes/variants for functional follow-ups. We noted the relatively small overlap of results from TWAS and colocalization. This might be because these methods assume the genetic architecture of both the trait of interest and the tissue gene expression differently. In addition, we observed the discrepancy between high *rcp* values and lack of correlation of raw *P*-values of GWAS and eQTL in the entire region of each colocalized locus. This may be due to 1) the allelic heterogeneity and complex LD in each locus; 2) the imperfect LD match between GWAS (only Holstein population) and eQTLs populations (multiple breeds); 3) the currently commonly used colocalization methods based on GWAS summary statistics might not work well in highly related individuals in livestock. We therefore suggest focusing analyses on loci where colocalization and TWAS methods agree.

Further integration of these QTLs with functional annotations from the Functional Annotation of Animal Genomes (FAANG) project will provide opportunities to understand transcriptional/post-transcriptional regulatory mechanisms underpinning GWAS hits for agronomic traits^23^. The multi-tissue e/sQTLs generated here will also enable the exploration of molecular mechanisms underlying the extensive pleiotropic effects identified in livestock^24^. This information will allow the understanding of mechanisms of response to intended selection as well as disentangling negative correlated responses to this same selection (e.g. increasing mastitis or deteriorating fertility when selection for increased milk production). Furthermore, this resource will assist in the development of genomic selection methods and tools to improve animal health and wellbeing. For instance, a better understanding of the genetic architecture underpinning agronomic traits will benefit genetic improvement programs by incorporating biological knowledge into genomic prediction models, which has been shown to improve prediction accuracy across populations and breeds^10,24^.

## Online Methods

### Ethics

The ethical approval for this project was obtained from the US Department of Agriculture, Agricultural Research Service, Beltsville Agricultural Research Center’s Institutional Animal Care and Use Committee (Protocol 16-016).

### Quantification of gene expression

We downloaded 11,642 RNA-Seq datasets (by June 24th, 2019) from SRA (n = 11,513, https://www.ncbi.nlm.nih.gov/sra/) and BIGD databases (n = 129, https://bigd.big.ac.cn/bioproject/) by searching the ‘Organism’ for ‘Cattle’ and the ‘Strategy’ for ‘RNA seq’. We merged multiple datasets from single samples, yielding 8,536 unique RNA-Seq samples. We applied a stringent and uniform pipeline to filter and analyze all the data. Briefly, we first removed adaptors and low quality reads using Trimmomatic (v0.39)^25^ with parameters: adapters/TruSeq3-SE.fa:2:30:10 LEADING:3 TRAILING:3 SLIDINGWINDOW:4:15 MINLEN:36. We filtered out samples with clean read counts ≤ 500K, resulting in 7,680 samples, and mapped clean reads to the ARS-UCD1.2 cattle reference genome^17^ using single or paired mapping modules of STAR (v2.7.0) with parameters of outFilterMismatchNmax 3, outFilterMultimapNmax 10 and outFilterScoreMinOverLread 0.66. We kept 7,264 samples with uniquely mapping rates ≥ 60% (mean, 91.07%; range, 60.44%-100%; mapping details in Supplementary Table 1). We then obtained normalized expression (TPM) of 27,608 Ensembl (v96) annotated genes using Stringtie (v2.1.1)^26^, and extracted raw read counts of them with featureCounts (v1.5.2)^27^. We finally clustered 7,264 samples based on log2(TPM +1) using a hierarchical clustering method, implemented in R (v3.4.1) package *dendextend*, with distance = (1-*r*), where *r* is the Pearson correlation coefficient.

### Quantification of alternative splicing

We used Leafcutter (v0.2.9)^28^ to identify and quantify variable alternative splicing events of genes by leveraging information of junction reads (i.e., reads spanning introns) that were obtained from the STAR alignment. The Leafcutter enables the identification of splicing events without relying on existing annotations that are typically incomplete, especially in the setting of large genes or individual- and/or population-specific isoforms^28^. We first converted bam files from STAR alignment into junction files using the script “bam2junc.sh”, and then performed intron clustering using the script “leafcutter_cluster.py” with default settings of 50 reads per cluster and a maximum intron length of 500 kb. We employed the “prepare_genotype_table.py” script in Leafcutter to calculate intron excision ratios and to remove introns used in less than 40% of individuals or with no variation. Ultimately, we standardized and quantile normalized intron excision ratios as Percent Spliced-In (PSI) values across samples. We clustered 7,180 samples based on PSI using the same method as used in gene expression.

### Genotyping and imputation

We called genotypes of known genomic variants in the 1000 Bull Genomes Projects^10^ for 7,180 high-quality RNA-Seq samples individually, following the recommended best practices pipeline in Genome Analysis Toolkit, (GATK) (v4.0.8.1)^29^ with default settings. We filtered out low quality SNPs using --filter-expression “FS > 30.0 & QD < 2.0”. We then imputed the filtered SNPs on autosomes to sequence level using Beagle (v5.1)^30^ based on a multiple-breed reference population consisted of 3,103 individuals from run7 of the 1000 Bull Genomes Project^10^ and 207 public individuals from *Bos taurus* (n = 101), *Bos indicus* (zebu, n = 20), and *Bos grunniens* (yak, n = 86) (Supplementary Table 15). Finally, we obtained 6,123 samples that were genotyped and imputed successfully. We filtered out variants with MAF < 0.05 and dosage R-squared (DR^2^) < 0.8, resulting in 3,824,444 SNPs used for QTL mapping. To evaluate the accuracy of imputation, we called genotypes (~6 M SNPs) from WGS (average read depth > 10×) of Holstein (n = 4), Limousin (n = 3) and Angus (n = 5) animals, which had RNA-Seq data as well. We then measured the genotype concordance rates between WGS-SNPs and RNA-Seq/imputed SNPs. We extracted 153,913 LD-independent SNPs using plink (v1.90)^31^ (--indep-pairwise 1000 5 0.2), and conducted PCA analysis for all 6,123 samples using these SNPs in EIGENSOFT (v7.2.1)^32^. We calculated the identity-by-state (IBS) distance among samples by using these independent SNPs to remove duplicate individuals. IBS distance = (IBS2 + 0.5*IBS1) / (IBS0 + IBS1 + IBS2), where IBS0 is the number of IBS 0 non-missing variants, IBS1 is the number of IBS 1 non-missing variants and IBS2 is the number of IBS 2 non-missing variants. We set an IBS distance cutoff of 0.85 to deem two samples as duplicates and kept one of them. When conducting QTL mapping, we removed an average of 43 duplicate samples within each tested tissue (ranging from one in salivary gland and leukocyte to 132 in muscle), resulting in 4,889 samples.

### Allele specific expression (ASE)

We conducted ASE analysis using the GATK ASEReadCounter tool (v4.0.8.1) with the following settings: --U ALLOW_N_CIGAR_READS -minDepth 10 -minMappingQuality 255 --minBaseQuality 10. SNPs for ASE detection fulfilled the following criteria: heterozygous in at least five samples, at least 10 reads per allele, and at least 2% of all reads supporting the minor allele. We then calculated a binominal *P*-value by comparing to the expected ratio under the null hypothesis, followed by multiple-test correction with the Benjamini-Hochberg approach (FDR). SNPs with FDR < 0.05 were considered as significant ASE. We estimated the effect size (allele fold change, aFC) of regulatory variants at ASE loci using a haplotype-based approach implemented in phASER (v1.1.1)^33^.

### Bioinformatics analysis of WGBS data

For WGBS data analysis, we first used FastQC (v0.11.2) and Trim Galore v0.4.0 (-- max_n 15 --quality 20 --length 20 -e 0.1) to determine read quality and to filter reads with low quality, respectively. We then mapped clean reads to the same reference genome (ARS-UCD1.2) using Bismark software (v0.14.5)^34^ with default parameters. After deduplication of reads, we extracted methylation levels of cytosines using the *bismark_methylation_extractor* (--ignore_r2 6) function. The coverages of all WGBS data were calculated using clean reads with an average of 27.6-fold coverage (range: 5-47 ×). Ultimately, we kept CpG sites that were represented by at least five reads for subsequent analyses. We visualized sample clusters based on DNA methylation levels of shared CpGs using *t*-SNE approaches.

### Identification of TAD and significant Hi-C contacts

To find potential chromatin interactions between distant eVariants and target eGenes, we identified TADs and Hi-C contacts from Hi-C data from lung tissue in cattle that was retrieved from NCBI Sequence Read Archive (SRA) under accessions: SRR5753600, SRR5753603, and SRR5753606. We used Trim Galore (v0.4.0) to trim adapter sequences and low-quality reads (--max_n 15 --quality 20 --length 20 -e 0.1), resulting in ~820 million clean reads. We then mapped clean reads to the reference genome (ARS-UCD1.2) using BWA(v0.7.17)^35^. We applied HiCExplorer v3.4.1^36^ to build a Hi-C contact matrix with 10kb resolution and identified TAD with hicFindTAD. We kept TADs with FDR less than 0.01 to link eQTLs to eGenes. We further employed HiC-Pro (v2.11.4)^37^ to call Hi-C contacts with 10 kb resolution from Hi-C data. Briefly, HiC-Pro aligned clean reads to the reference genome with Bowtie2 (v2.3.5)^35,38^. After building a contact matrix, HiC-Pro generated intra- and inter-chromosomal maps and normalized them using the ICE normalization algorithm. We converted Hi-C contact matrix in HiC-Pro format to FitHiC format using HiCPro2FitHiC.py in FitHiC (v2.0.7) and applied statistical confidence estimates to determine the significant intra-chromosome contacts (Benjamini-Hochberg corrected *P* < 0.05).

### Tissue-specificity analysis of gene expression, alternative splicing and DNA methylation

To quantify tissue-specific expression of genes, we computed a *t*-statistics for each gene in each of the 114 tissues. We grouped 114 tissues into 13 categories (Supplementary Table 1). We scaled the log2-transformed expression (i.e., log_2_TPM) of genes to have a mean of zero and variance of one within each tissue. We then fitted a linear model as described in^15^ for each gene in each tissue using the least squares method. When constructing the matrix of dummy variables (i.e., design matrix) for tissues, we denoted samples of the target tissue/cell type (e.g., CD4 cells) as ‘1’, while samples outside the target category (e.g., non-blood/immune tissues) as ‘-1’. We excluded samples within the same category (e.g., CD8 cells and lymphocytes) to detect genes with specific expression in each particular category, even if they were not specific to the target tissue within this category. We obtained *t*-statistics for each gene to measure its expression specificity in a given tissue. We considered the top 5% of genes ranked by largest *t*-statistics as genes with high tissue-specific expression. In order to explore the conservation of tissue-specific expression between cattle and humans, we employed the same method to quantify the tissue-specific expression of all orthologous genes in each of 55 human tissues using GTEx (v8) data^3^.

To detect tissue-specific alternative splicing, we used leafcutter to analyze the differential intron excision by comparing the samples from the target tissue to the remaining tissues^28^, while excluding samples from tissues of the same category as the target tissue. We used the Benjamini-Hochberg method (FDR) to control multiple testing.

For DNA methylation, we focused on gene promoters (from upstream 1500bp to downstream 500bp of TSS based on the ARS-UCD1.2 from Ensembl v99), the methylation levels of which were calculated with a weighted methylation method using the roimethstat function in MethPipe (v3.4.3)^39^. We computed a *t*-statistic for the promoter of each gene using the same method as in tissue-specific expression analysis. We considered the bottom 5% of genes ranked by *t*-statistics as genes with tissue-specific promoter hypomethylation. We also detected tissue-specific methylation regions in a genome-wide mode using SMART2 (v2.2.8)^40^ with parameters of -t DeNovoDMR -MR 0.5 -AG 1.0 -MS 0.5 -ED 0.2 -SM 0.6 - CD 500 -CN 5 -SL 20 -PD 0.05 -PM 0.05.

### Covariate analysis for QTL discovery

To account for hidden batch effects and other technical/biological sources of transcriptome-wide variation in gene expression, we estimated latent covariates in each tissue using the Probabilistic Estimation of Expression Residuals (PEER v1.3) method^41^. In each tissue, we estimated 75 PEER factors first. The posterior variances of factor weights dramatically decreased and reached or nearly reached plains when 10 PEER factors were included (Extended Data Figure 6a). Therefore, we used 10 PEER covariates to account for the effects of confounding variables on gene expression in all following QTL analyses. For instance, the variance of gene expression among samples in adipose captured by 9 out of 10 PEER factors were significantly (FDR < 0.05) correlated with known technical and biological covariates like clean data size, mapping rate, project, breeds, sub-species, sex and age (Extended Data Figure 6b). To further control the effect of population structure on the discovery of QTLs, we included genotype PCs based on sample size bins: three PCs for tissues with < 150 samples, five PCs for tissues with ≥ 150 and < 250 samples, and ten PCs for tissues with ≥ 250 samples.

### *cis*-eQTL mapping

We conducted *cis*-eQTL mapping for 23 distinct tissues with at least 40 individuals each, while adjusting for corresponding PEER factors and genotype PCs. Detailed information about these 23 distinct tissues is in Supplementary Table 4. As the majority of *cis*-eQTLs are shared across sub-species/breeds (Figure 3g), we combined, adjusting for species/breed, all of the datasets from the same tissue to perform cis-eQTL mapping in order to increase the statistical power. We kept genes with TPM > 0.1 in ≥ 20% samples in each tissue. Gene expression values of all samples in a given tissue were quantile normalized to the average empirical distribution and expression values for each gene then inverse normal transformed (INT) across samples. The *cis*-eQTL mapping was done using a linear regression model, implemented in FastQTL (v2.184)^42^, to test associations of the normalized expression level of genes with genetic variants in 1Mb of TSS of target genes. We only considered imputed variants with MAF > 0.05 and at least four minor alleles across samples within the target tissue. We first conducted *cis*-eQTL mapping in a permutation mode with the setting --permute 1000 10000, to identify genes with at least one significant *cis*-eQTL (eGene). We considered FDR ≤ 0.05 as significant, which was calculated with the Benjamini-Hochberg method based on the beta distribution-extrapolated empirical *P*-values from FastQTL. To identify a list of significant eGene-eVariant pairs, we applied the nominal mode in FastQTL. A genome-wide empirical *P*-value threshold *p_t_* was defined as the empirical *P*-value of the gene closest to the 0.05 FDR threshold^3^. We then calculated the nominal threshold as *F*^-1^(*p_t_*), where *F^-1^* is the binominal inverse cumulative distribution, of which parameters for genes were obtained from the above permutation mode of FastQTL analysis. We considered variants with nominal *P*-values below the nominal threshold as significant, and included them into the list of eGene-eVariant pairs. We calculated the aFC, defined as the ratio of the expression level of the haplotype carrying the alternative allele over the one carrying the reference allele, to measure effect sizes of *cis*-eQTLs using the aFC (v0.3) tools^43^. We further applied the statistical fine-mapping method, dap-g (v1.0.0)^44^, to infer multiple independent casual *cis*-eQTLs of a gene in a tissue. The dap-g approach employed a Bayesian variable selection model, using a signal-level posterior inclusion probability (SPIP) to measure the strength of each association signal (SNPs in LD). We set a cutoff of 0.1 (i.e., SPIP > 0.9) as the inclusion threshold to detect representative/independent eQTLs for the target eGene. To analyze pairwise tissue similarity in QTLs, we calculated π1 statistics, defined as the proportion of true positive QTLs identified in first tissue (Discovery tissue) amongst all tested gene-variant pairs in second tissue (Validation tissue), using the Storey and Tibshirani qvalue approach, as described in^13^.

### Meta-analysis of *cis*-eQTLs of muscle samples from three sub-species

Data from muscle samples were available from three sub-species: *Bos indicus* (n = 51), *Bos taurus* (n = 505), and their crosses (n = 108). To explore the similarity and variability of *cis*-eQTLs among sub-species, we conducted *cis*-eQTL mapping using muscle samples from each of the sub-species separately. We then conducted a meta-analysis to integrate *cis*-eQTL results from three sub-species using the METAL (v2020-05-05) tool^45^. We obtained Z-scores (the sum of weighted effect sizes) of SNPs from the meta-analysis. Weights were proportional to the square-root of the number of individuals in each sub-species^45^. We employed plink (v1.90)^31^ to test the SNP × breed interaction in muscle samples, and adjusted the *p*-values to FDR using Benjamini-Hochberg procedure. We took FDR < 0.05 as the significant threshold.

### *cis*-sQTL mapping and tissue-sharing patterns

In each of the 23 distinct tissues, we applied a linear regression model, implemented in FastQTL^42^, to test for associations of genotypes within 1 Mb up- and down-stream of target intron clusters and their corresponding intron excision ratios. We used the first five genotype PCs to account for the effect of ancestry, and 10 PEER factors to adjust for the effect of unknown confounding variables. We applied the permutation pass mode (--permute 1000 10000) in FastQTL^42^ to obtain beta approximated permutation *P* values, followed by multiple test correction with the FDR method. We considered sQTL-intron pairs with FDR < 0.05 as significant, and defined sGene as genes containing a significant sQTL in any introns. We employed MashR (v0.2.57)^46^ to analyze tissue-sharing patterns of QTLs^3^, and considered the local false sign rate (LFSR) < 0.05 as significant.

### *trans-QTL* mapping

We conducted *trans*-eQTLs for 15 tissues with at least 100 samples each. We filtered genomic variants using a more stringent threshold than *cis*-eQTL mapping to partially account for the reduction in statistical power. We obtained mappability of variants based on k-mer lengths of 36 and 75 following the procedure described in https://wiki.bits.vib.be/index.php/Create_a_mappability_track. Briefly, we calculated the mappability of variants with 36 and 75 k-mer based on ARS-UCD1.2 using a fast mapping based algorithm^47^, allowing for 2 mismatches. For each gene, we averaged the mappability across exons with 72 k-mer length and UTRs with 36 K-mer length. We excluded any variants within repeats (Repeatmasker and simple repeats), and further removed variants with mappability < 1, based on k-mer length of 75. After filtering, we kept SNPs with MAF > 0.05 and at least 10 minor alleles within each tissue for association testing.

We applied two methods to detect *trans*-eQTLs for protein-coding genes with an average mappability ≥ 0.8. Firstly, we associated the normalized expression of target genes with genotypes on other autosomal chromosomes using a linear regression model in MatrixQTL (v2.3)^48^, while adjusting for the same covariates as in *cis*-eQTL analysis. We further removed *trans-eQTL-gene* pairs that were cross-mappable to reduce false positives^49^. Secondly, we employed a linear mixed model (by fitting a polygenic effect with the genetic relationship matrix to further account for the complex relatedness among individuals) in the GCTA (v1.93.3beta)^50^ for *trans-eQTL* and *trans*-sQTL mapping. For both methods, we adjusted *P*-values for multiple testing using the Benjamini-Hochberg method to obtain FDR. We considered gene-variant pairs with FDR < 0.05 as significant. To conduct an internal validation of *trans*-eQTL mapping, we randomly and evenly divided blood and muscle samples into two groups. We conducted *trans*-eQTL mapping in the first group using the linear mixed model to detect significant *trans*-eQTL-gene pairs, and then repeated in the second group.

### TWAS and Colocalization of *cis*-eQTLs and GWAS loci

To associate gene expression in a tissue with complex traits, we conducted a single-tissue TWAS analysis using S-PrediXcan (v0.6.1)^51^ by prioritizing GWAS summary statistics for 43 agronomic traits of economic importance in cattle (Supplementary Table 10), including reproduction (n = 11), production (milk-relevant; n = 6), body type (n = 17), and health (immune/metabolic-relevant; n = 9). For body conformation (type), reproduction, and production traits, we conducted a single-marker GWAS by fitting a linear mixed model in 27,214 U.S. Holstein bulls^18^. For health traits, we conducted GWAS using the same method in a subset (ranging from 11,880 for hypocalcemia to 24,699 for livability) of the 27,214 available bulls^19^. We constructed a Nested Cross Validated Elastic Net prediction model using genotype and expression data. We included sub-species, 10 PEER factors and corresponding genotype PCs in the model to adjust for unknown confounding variables and underlying population structure. For each trait, we conducted TWAS in each of the same 23 distinct tissues as in *cis*-eQTL mapping. We considered genes with Bonferroni-corrected *P* < 0.05 as significant. We visualized the Manhattan plots of *P*-values of all tested genes using ggplot2 (v3.3.2) in R (v3.4.1). In addition, we further employed S-MultiXcan (v0.6.1)^52^ to conduct multi-tissue TWAS analysis, and considered gene-trait pairs with Bonferroni threshold *P* < 4×10^-6^ (0.05/13,024) significant.

To detect the shared causal variants of gene expression and complex traits, we conducted a colocalization analysis of *cis*-eQTLs from 23 distinct tissues and GWAS loci of 43 agronomic traits using fastENLOC (v1.0)^53^. Briefly, we split the imputed GWAS summary statistics into approximately LD-independent regions, and each region was considered as a GWAS locus. The LD-independent regions were generated from genotypes of 886 Holstein animals from run7 of 1000 bull Genomes project, as the GWAS summary statistics were from the U.S. Holstein population. In each GWAS locus of a trait with suggestive significant SNPs (*P* < 10^-5^), we considered a gene with regional colocalization probability (*rcp*) > 0.5 as significant. We further conducted the colocalization analysis using Coloc (v5.1.0)^54^ with the function coloc.abf. We obtained posterior probability values for H4 case (PP.H4), i.e., both traits (GWAS trait and eQTLs) are associated and share a single causal variant. We kept the tissue-trait-gene triples with PP.H4 > 0.8 for downstream analysis.

### Other downstream bioinformatics analysis

We used Genomic Association Tester (GATv1.3.4)^55^ 1,000 permutations to estimate the functional enrichment of QTLs in particular genomic regions, e.g., chromatin states and methylation elements. We considered enrichments with FDR < 0.05 as significant. We used the R package, ClusterProfiler (v3.0.4)^56^, to annotate the function of genes based on the Gene Ontology database from Bioconductor (org.Bt.eg.db v3.11.4). We considered GO terms with FDR < 0.05 as significant.

### Statistics & Reproducibility

No statistical method was used to predetermine sample size. We used all data passing standard quality controls, resulting in 7180 samples. For RNA-seq samples, we filtered out samples with clean read counts ≤ 500K or uniquely mapping rates < 60%, resulting in 7,180 samples. For genotypes, we filtered out SNPs with MAF < 0.05 or imputation dosage R-squared (DR2) < 0.8, resulting in 3,824,444 SNPs used for QTL mapping. For the QTL mapping in each tissue, we set an identity-by-state (IBS) distance cutoff of 0.85 to deem two samples as duplicates and kept one of them for analysis. The details of data exclusions are available in the Methods section. For all the boxplots, horizontal lines inside the boxes show the medians. Box bounds show the lower quartile (Q1, the 25^th^ percentile) and the upper quartile (Q3, the 75^th^ percentile). Whiskers are minima (Q1 - 1.5 × IQR) and maxima (Q3 + 1.5 × IQR), where IQR is the interquartile range (Q3-Q1). Outliers were not shown in the boxplots. The experiments were not randomized, as all the datasets are publicly available and from observational studies. The Investigators were not blinded to allocation during experiments and outcome assessment, as the data are not from controlled randomized studies.

## Extended Data

**Figure:**
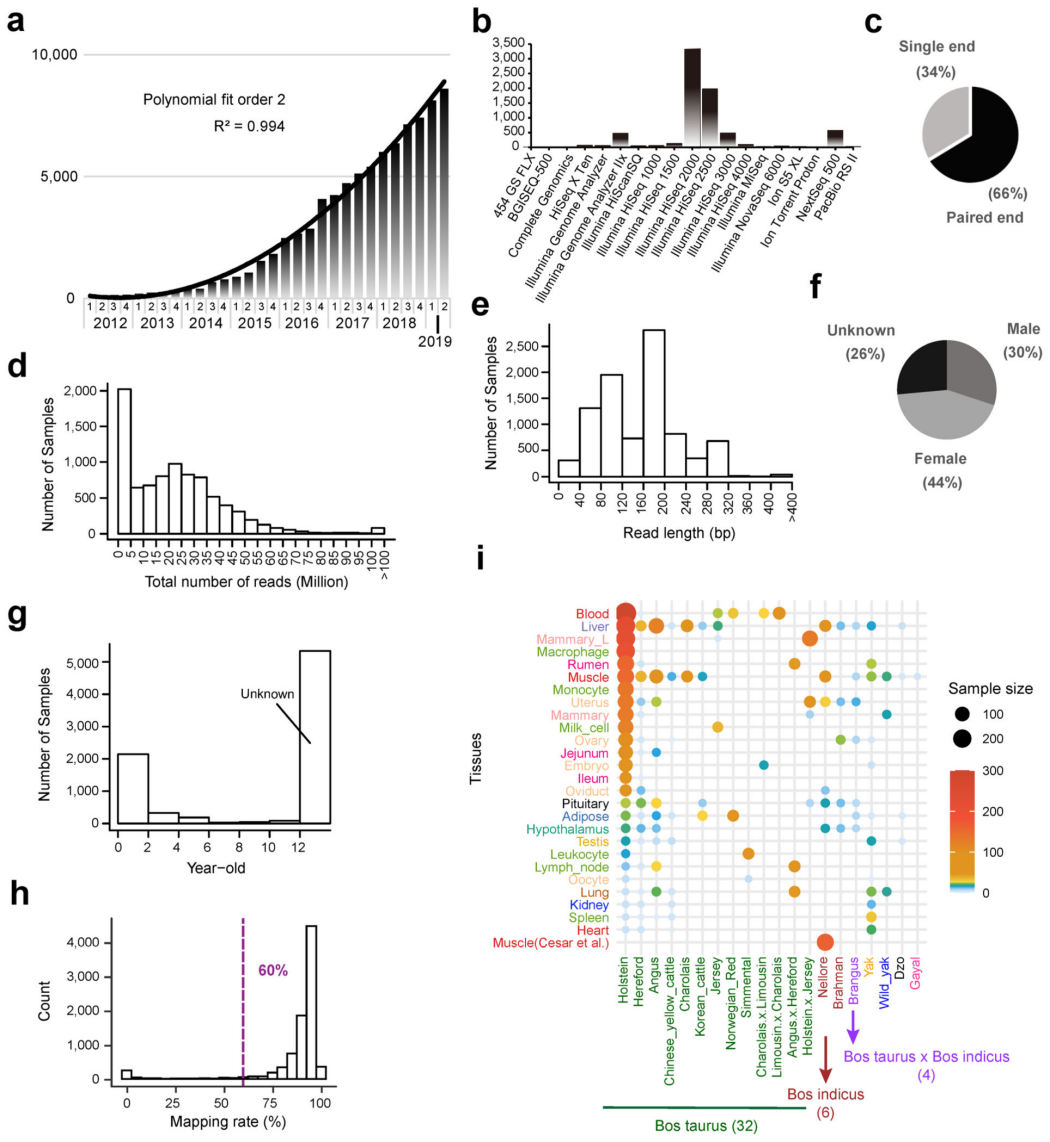


**Figure:**
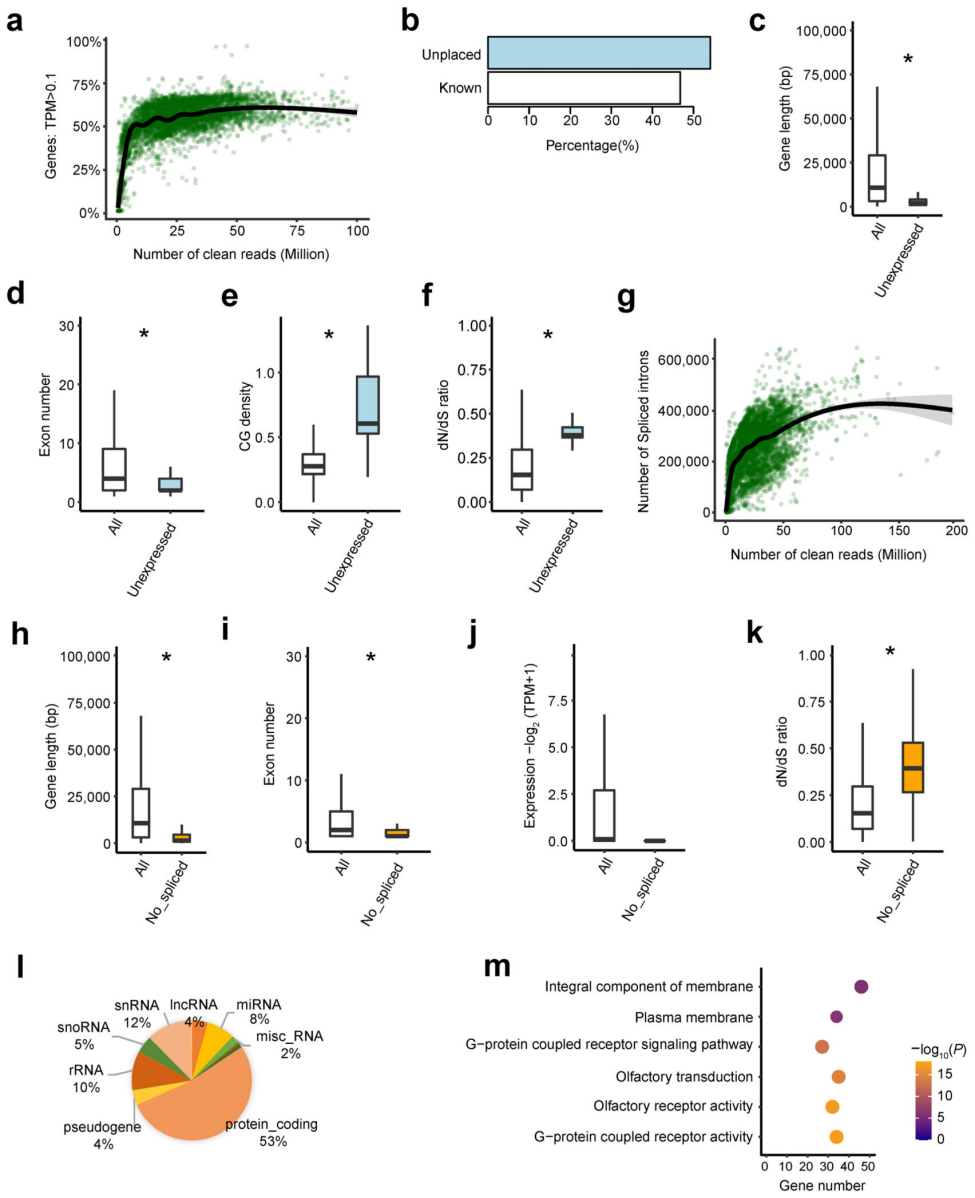


**Figure:**
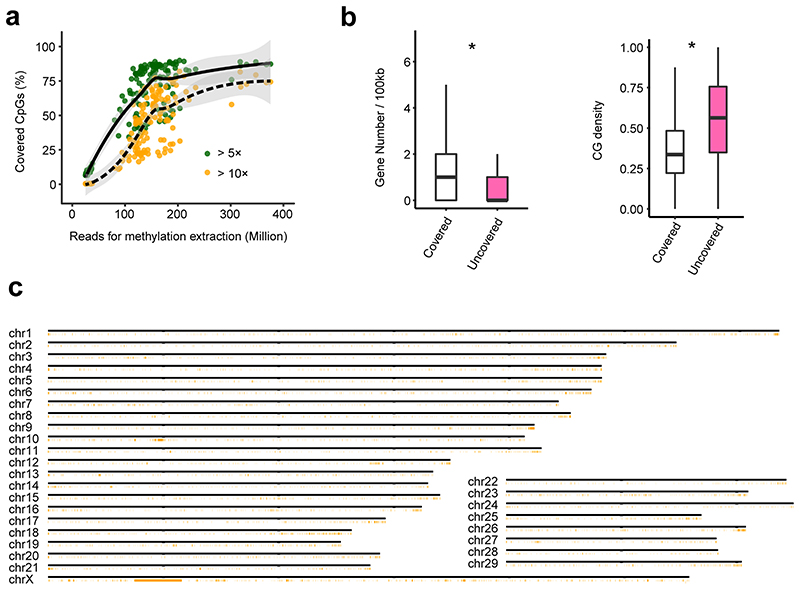


**Figure:**
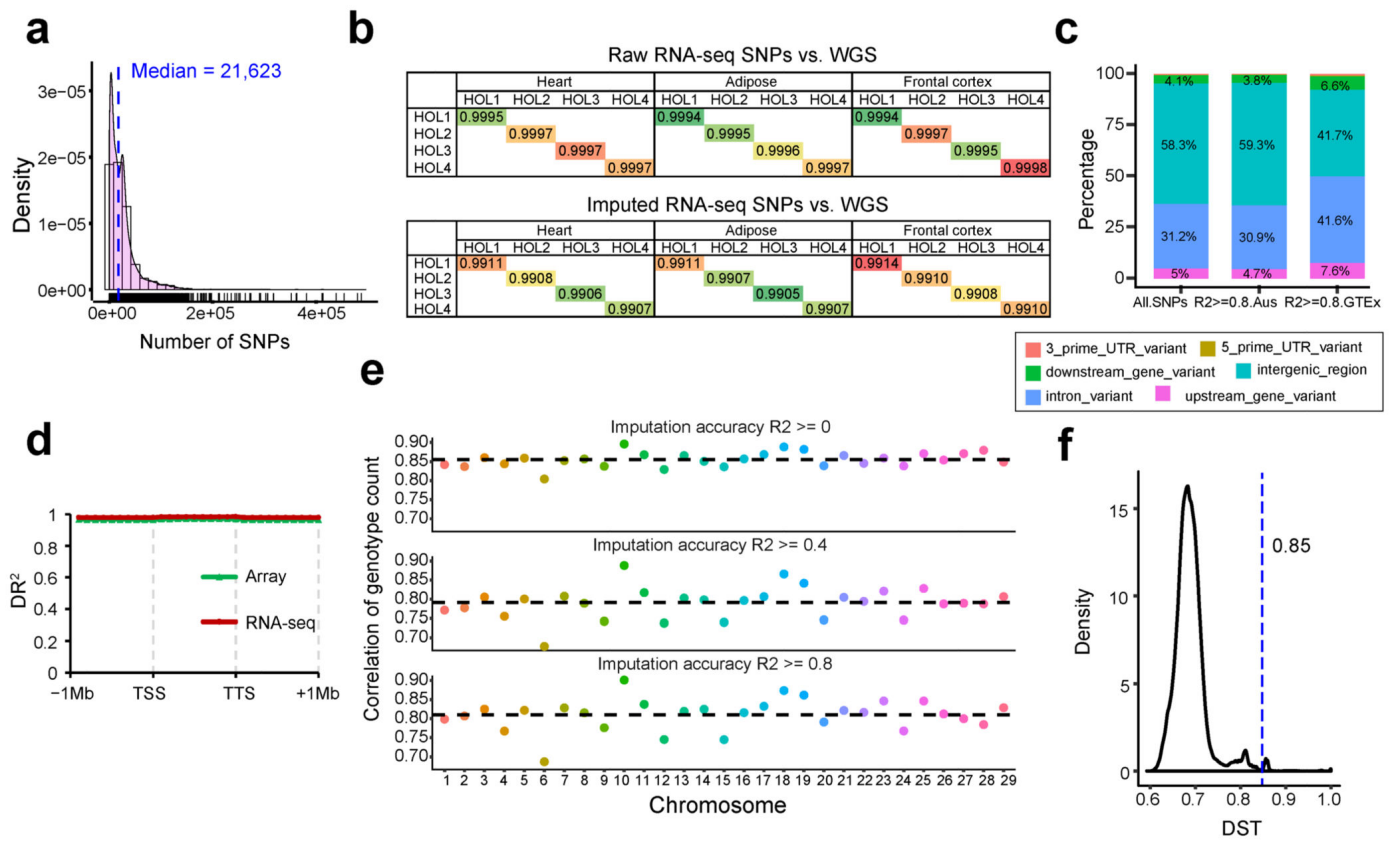


**Figure:**
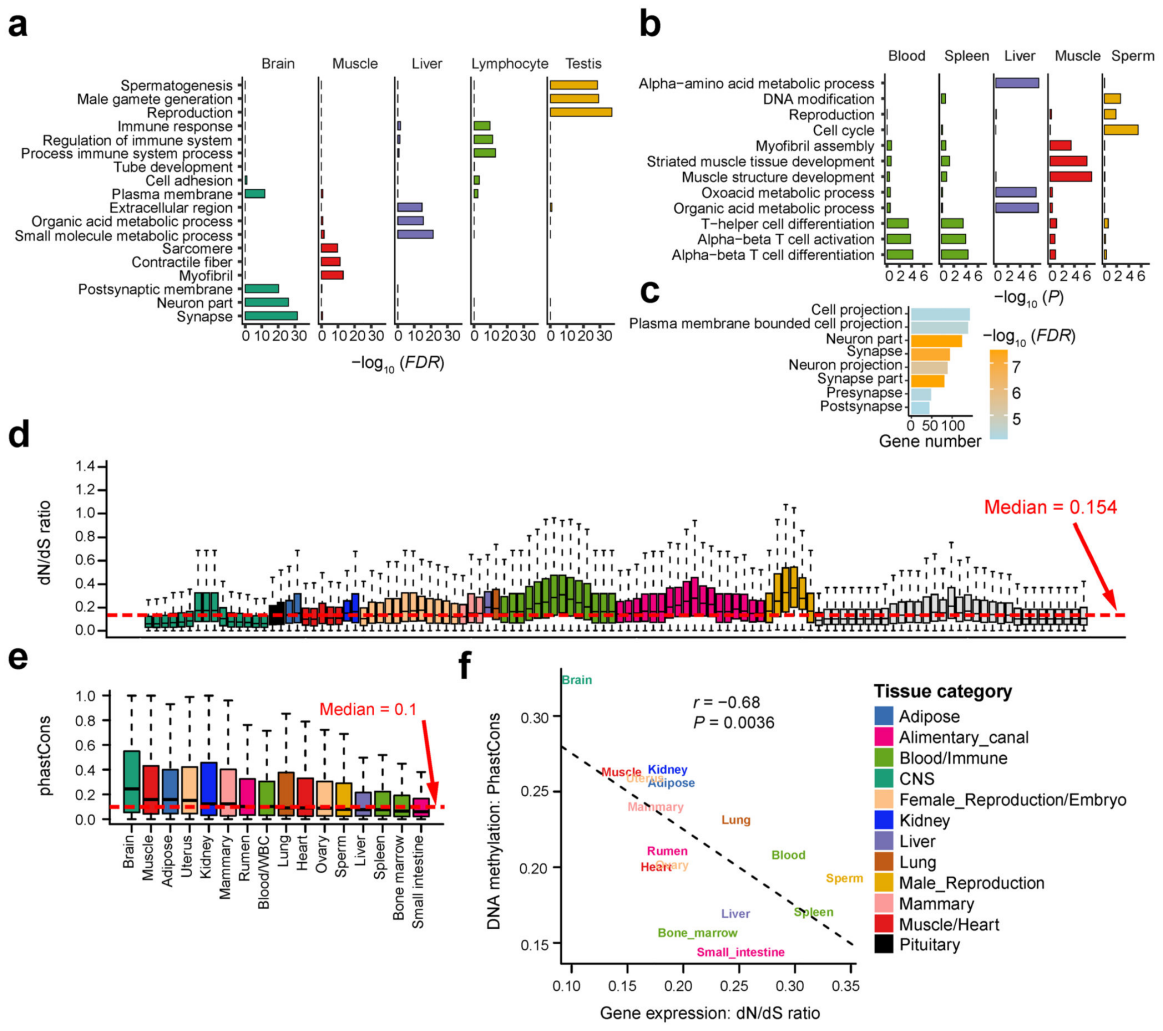


**Figure:**
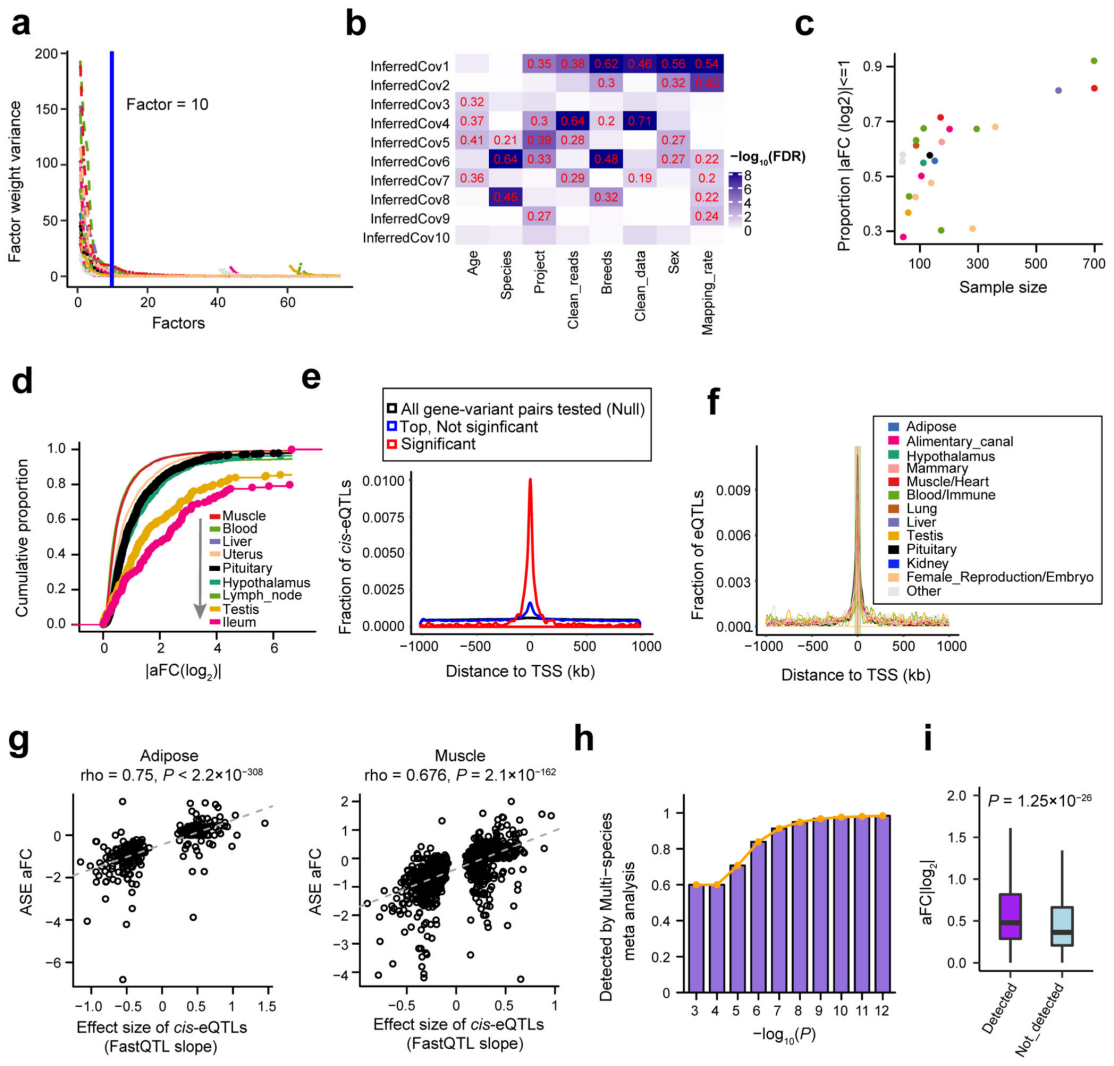


**Figure:**
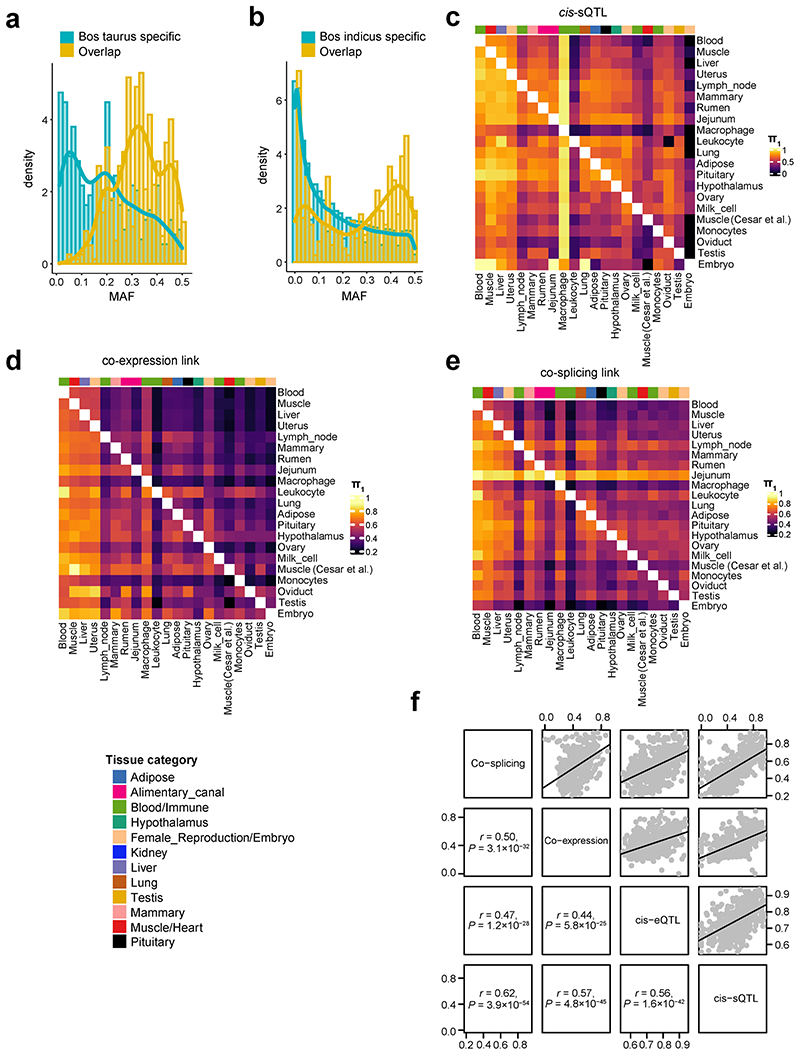


**Figure:**
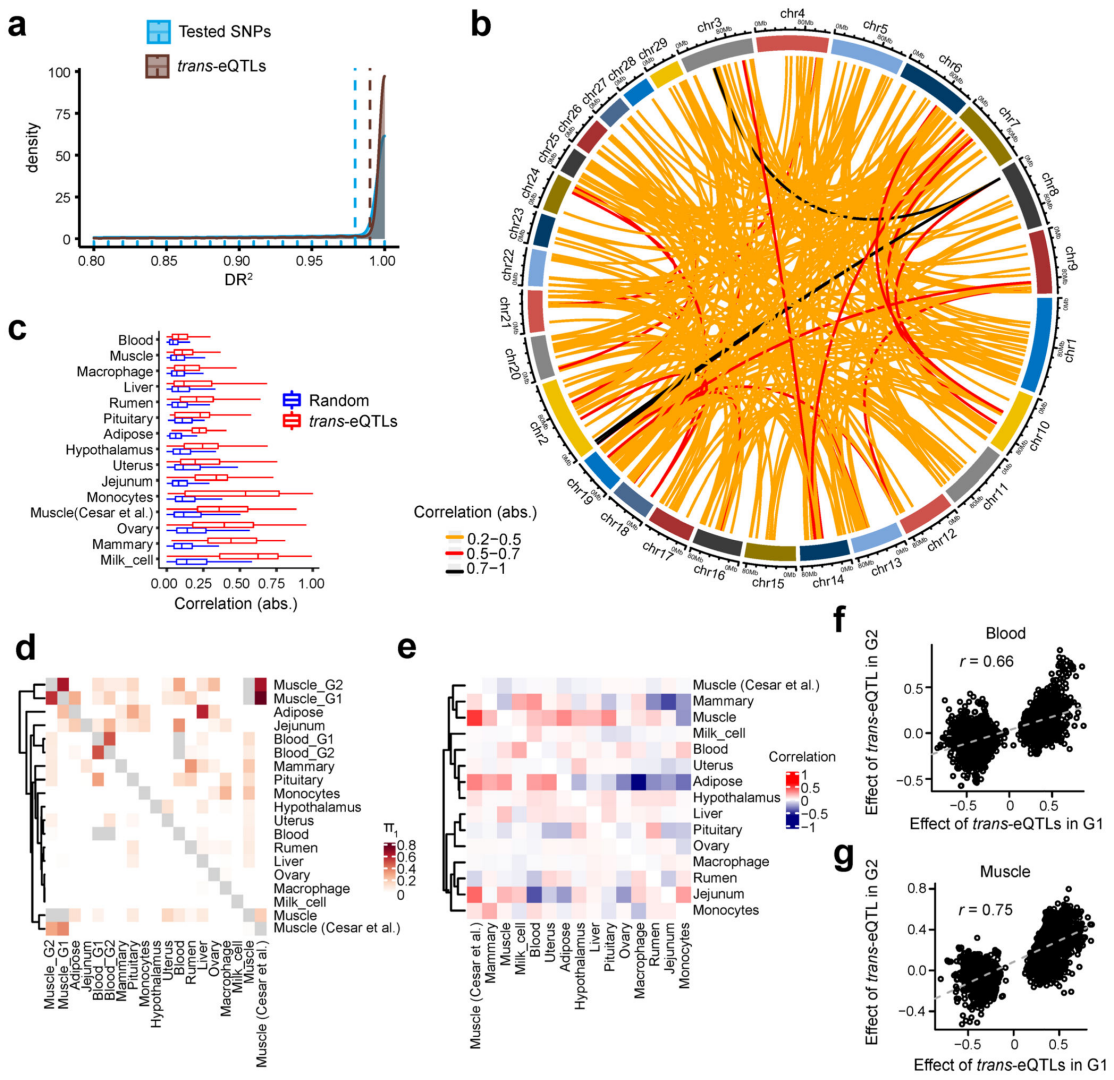


**Figure:**
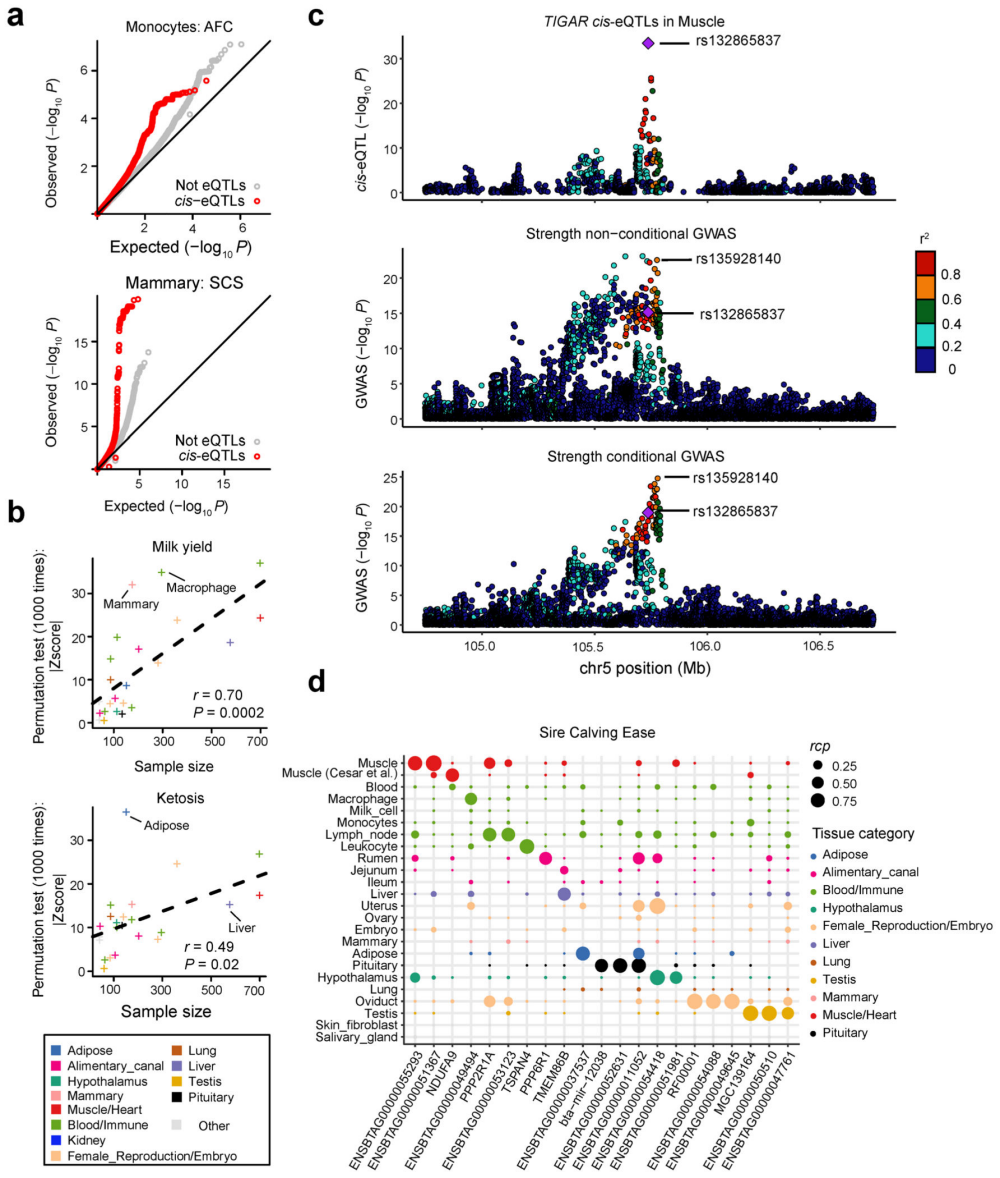


**Figure:**
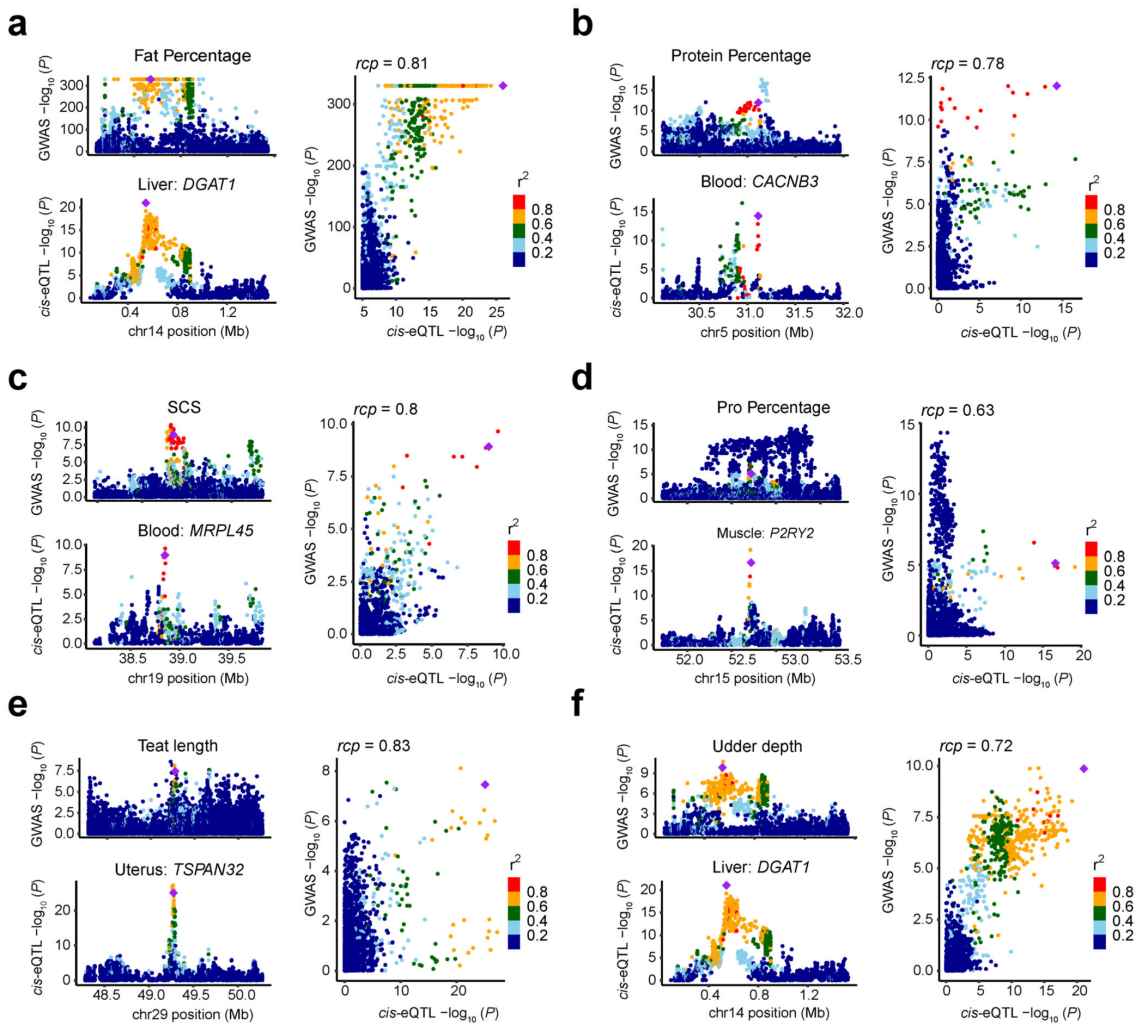


## Data Availability

All raw data analyzed in this study are publicly available for download without restrictions from SRA (https://www.ncbi.nlm.nih.gov/sra/) and BIGD (https://bigd.big.ac.cn/bioproject/) databases. Details of RNA-Seq, WGBS and WGS can be found in Supplementary Table 1, 2 and 15, respectively. All processed data, the full summary statistics of QTL mapping are available at https://cgtex.roslin.ed.ac.uk/.

## Code Availability

All the computational scripts and codes for RNA-seq and DNA methylation data quantification, quality control, gene expression normalization, genotype imputation, QTL mapping, functional enrichment, TWAS and colocalization are available at both the web portal of CattleGTEx (https://cgtex.roslin.ed.ac.uk/) and the_github website (https://github.com/shuliliu/cattleGTEx. DOI: 10.5281/zenodo.6510550)^57^
